# Supplementary material for: Cardiac myosin binding protein-C phosphorylation as a function of multiple protein kinase and phosphatase activities
Source: Nat Commun. 2024 Jun 14;15:5111. doi: 10.1038/s41467-024-49408-5 (PMC11178824; doi:10.1038/s41467-024-49408-5)
Supplement: Supplementary file 1 — Supplementary Information [file 41467_2024_49408_MOESM1_ESM.pdf]

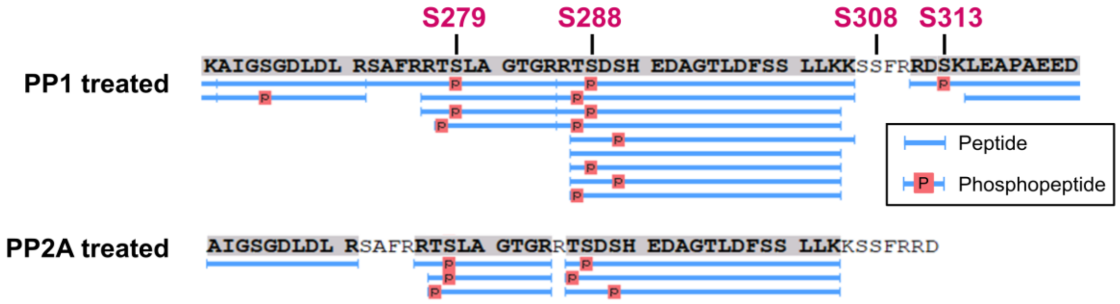

Supplementary Figure 1: Phosphopeptide specific mass spectrometry analysis of 2P-bands from PhosTag^{TM} SDS-PAGE gels. Clustering of phosphopeptides with multiple apparently different phosphor-residues adjacent to S279 and S288 is likely an artefact resulting from limited resolution. Phosphopeptides from each cluster likely correspond either to pS279 or pS288.

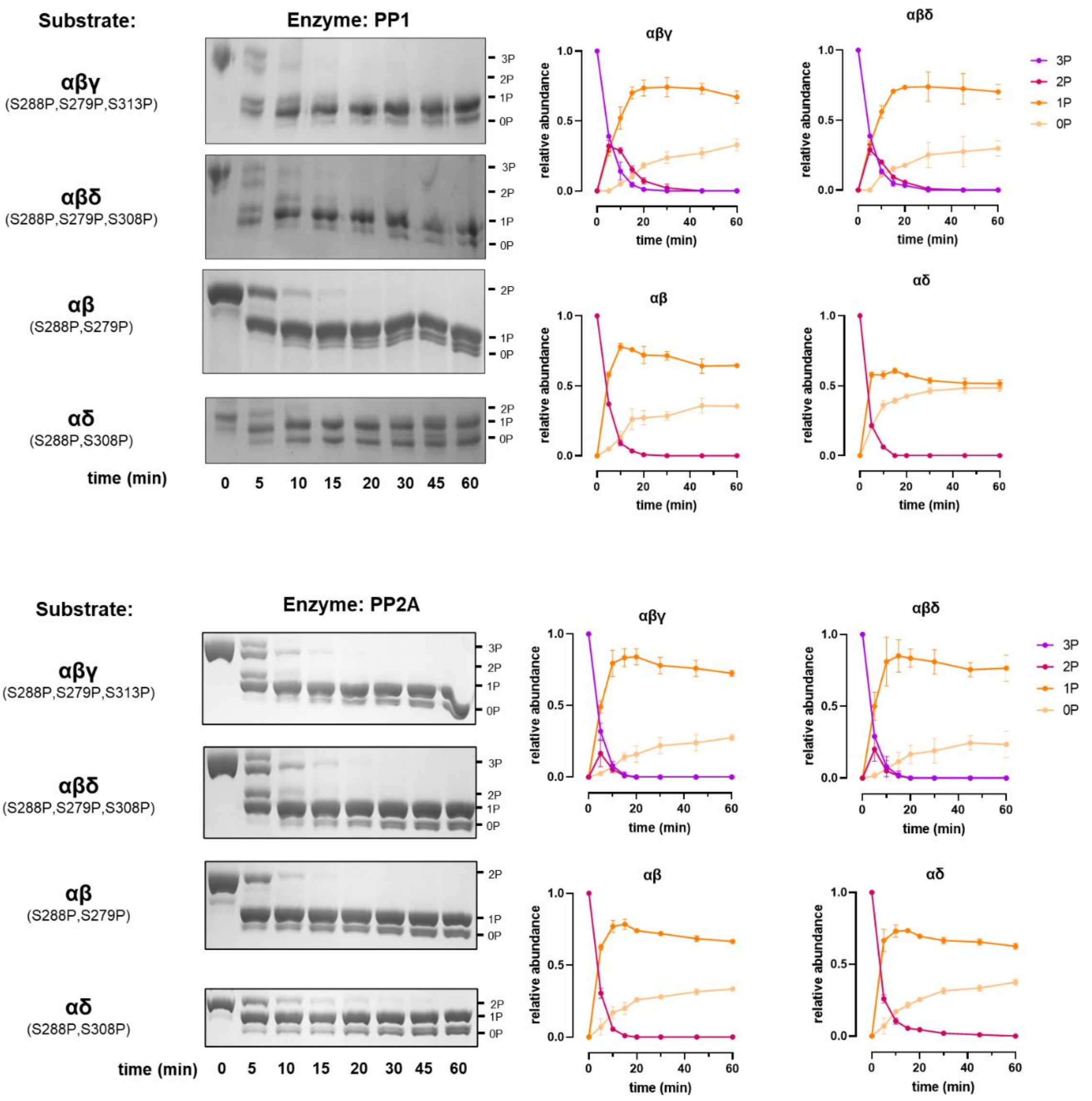

Supplementary Figure 2: Left: additional dephosphorylation time course experiments of various C1mC2 phosphoforms by phosphatases PP1 and PP2A visualized by PhosTag^{TM} SDS-PAGE gels. Right: Quantification of the data. Each datapoint represents the mean ± SD of n = 2 experiments.

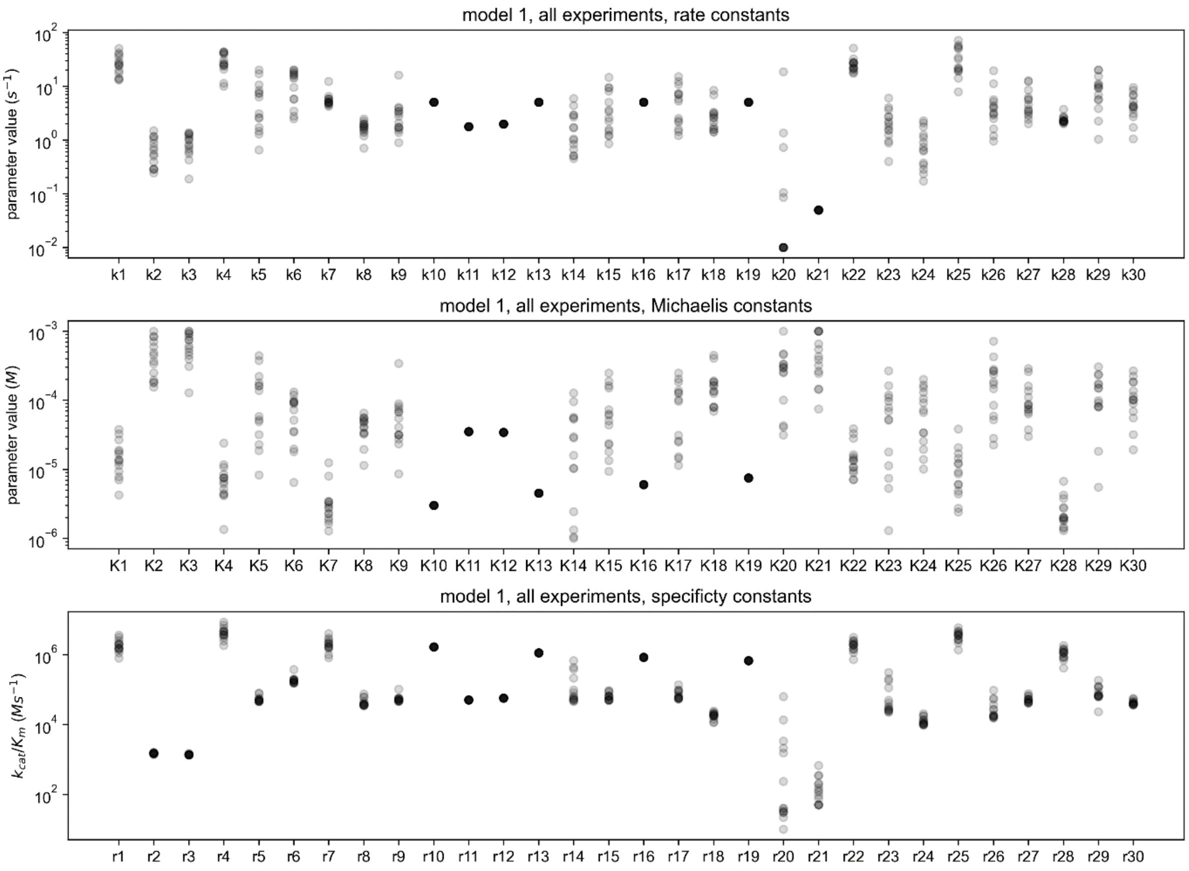

Supplementary Figure 3: Resulting parameters for model 1 after fitting the model to all data and filtering out poorly performing parameter sets (n=14).

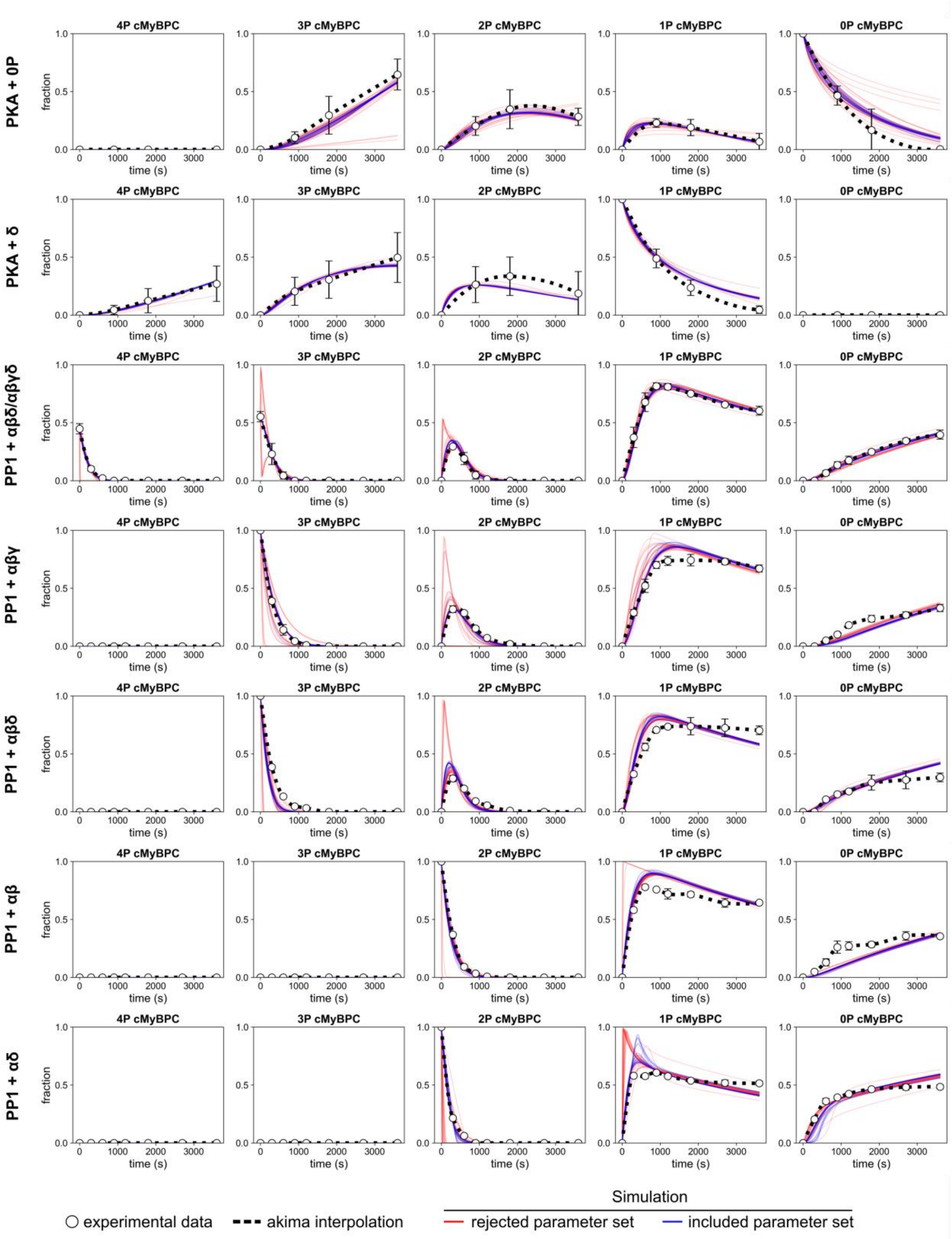

Supplementary Figure 4: Fit of model 1 to all data (n=50 parametersets, 14 included, 36 rejected), results shown for PKA and PP1 time course data. Each experimental data point represents the mean ± SD of n = 2-6 experiments.

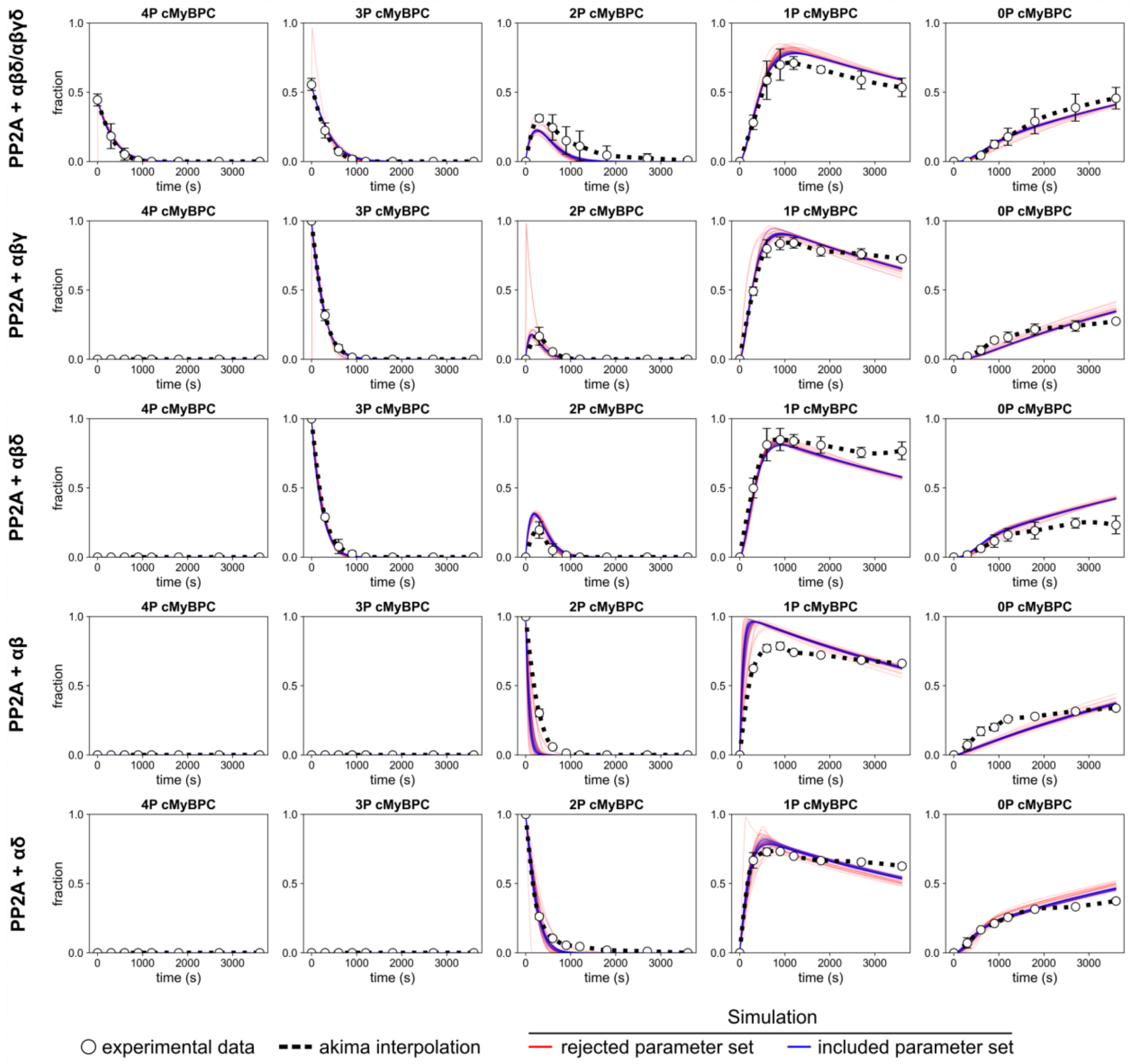

Supplementary Figure 5: Fit of model 1 to all data (n=50 parametersets, 14 included, 36 rejected), results shown for PP2A time course data. Each experimental data point represents the mean ± SD of n = 2-3 experiments.

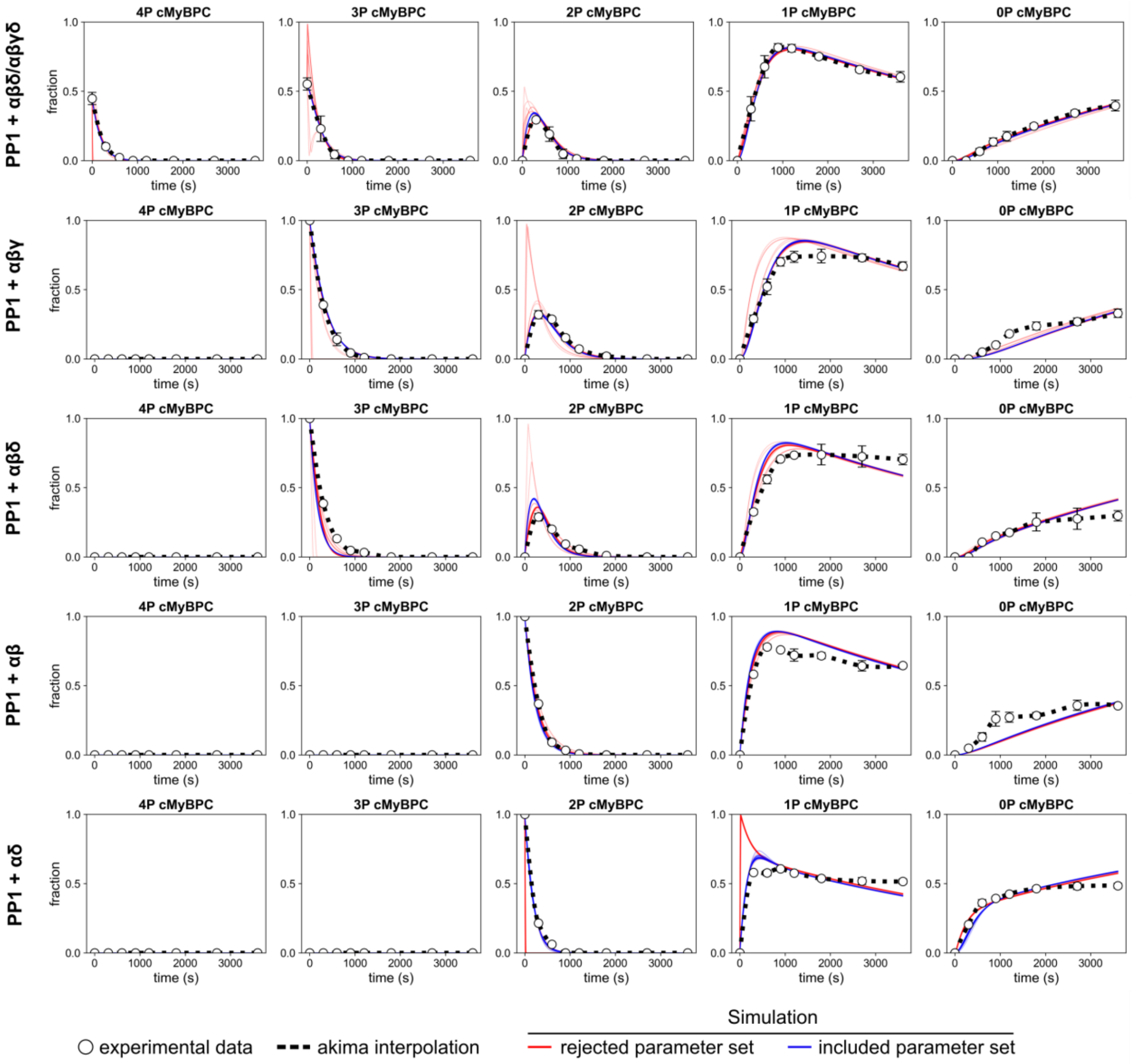

Supplementary Figure 6: Fit of model 1 to PP1 time course data only (n=50 parametersets, 23 included, 27 rejected). Each experimental data point represents the mean ± SD of n = 2-3 experiments.

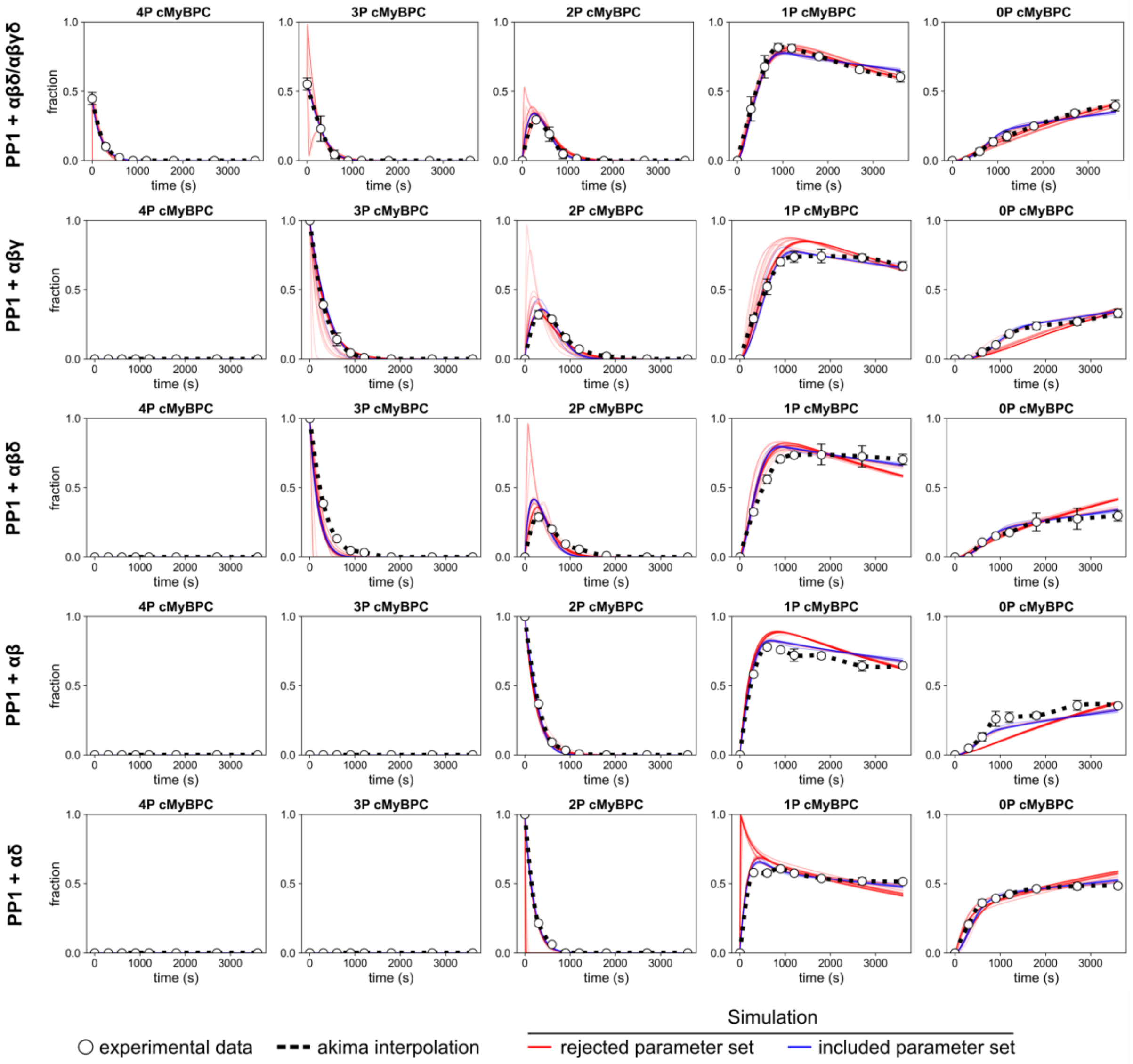

Supplementary Figure 7: Fit of model 2 (phenomenological model of direct activation of α dephosphorylation by 2P-C1mC2) to PP1 time course data only (n=50 parametersets, 9 included, 41 rejected). Each experimental data point represents the mean ± SD of n = 2-3 experiments.

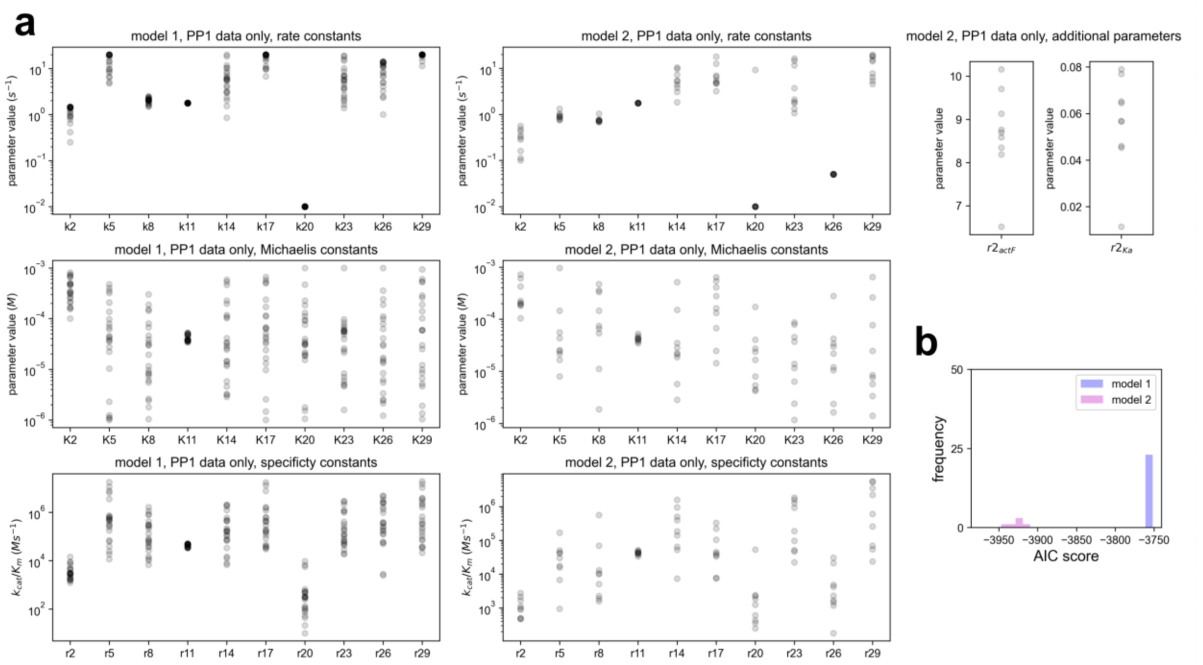

Supplementary Figure 8: **a**, Resulting parameters for model 1 and model 2 after fitting the models to PP1 data only and filtering out poorly performing parameter sets. **b**, Model comparison using the Akaike information criterion based on included parametersets after filtering (23 parameter sets included for model 1, 9 parameter sets included for model 2). Model 2 has a significantly lower AIC score than model 1 ($p = 1.6x10^{-5}$; Mann-Whitney test) and thus is to be preferred over model 1.

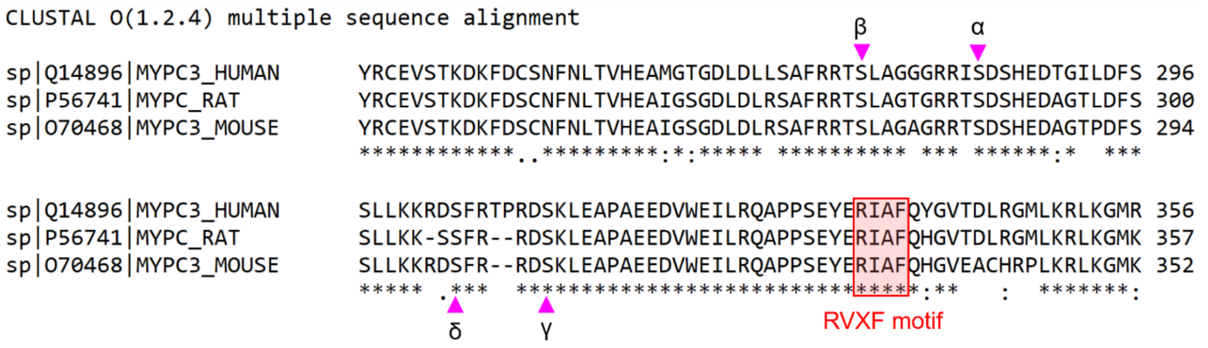

Supplementary Figure 9: Aligned cMyBP-C sequences from human, rat and mouse. Highlighted in red is the RVXF-motif (consensus sequence: [K/R][I/V][x][F/W]) which might enable binding between cMyBP-C and PP1. Pink arrowheads indicate phosphorylation sites.

`

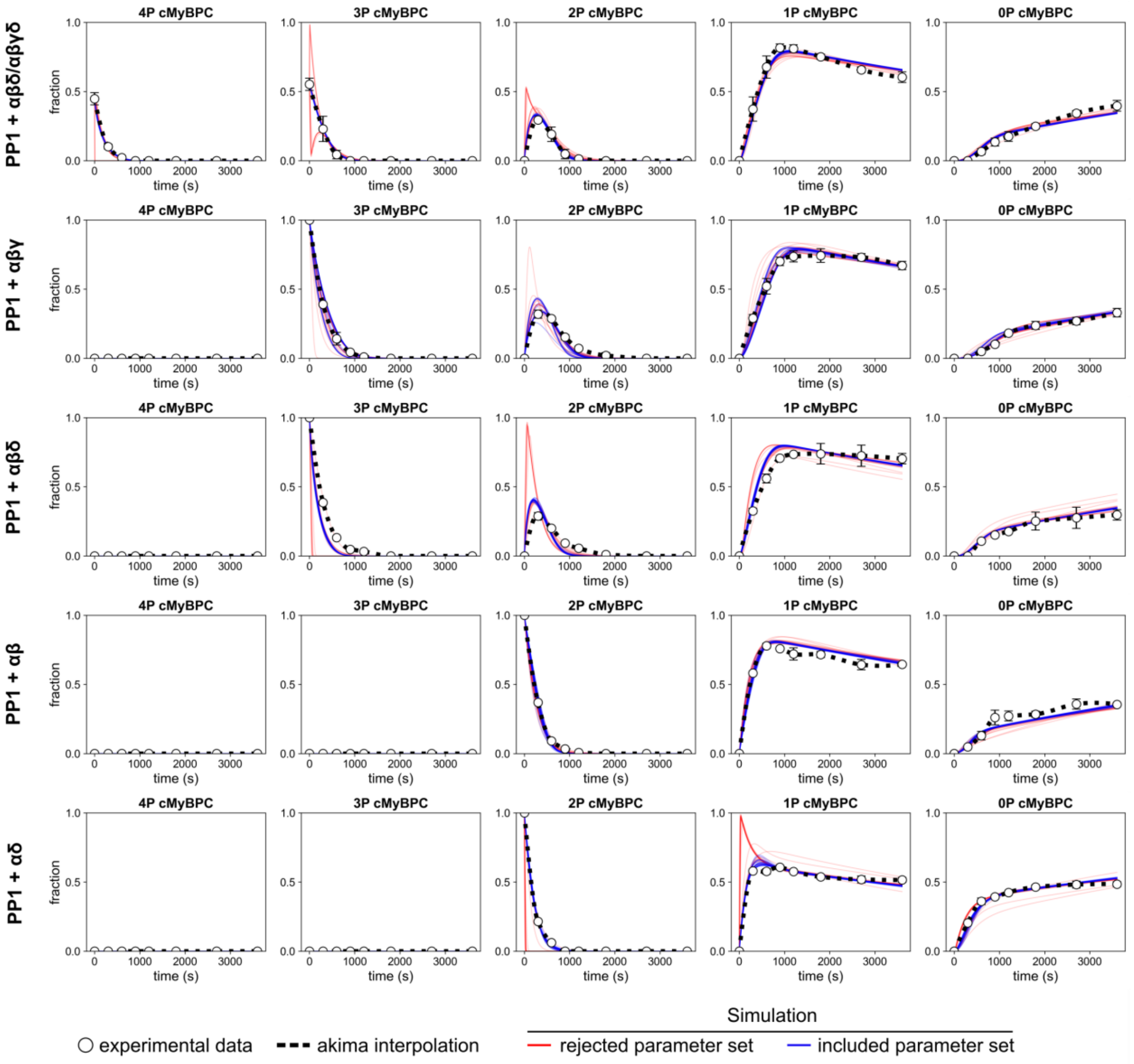

Supplementary Figure 10: Fit of model 3 (allosteric activation of PP1) to PP1 time course data only (n=50 parametersets, 24 included, 26 rejected). Each experimental data point represents the mean ± SD of n = 2-3 experiments.

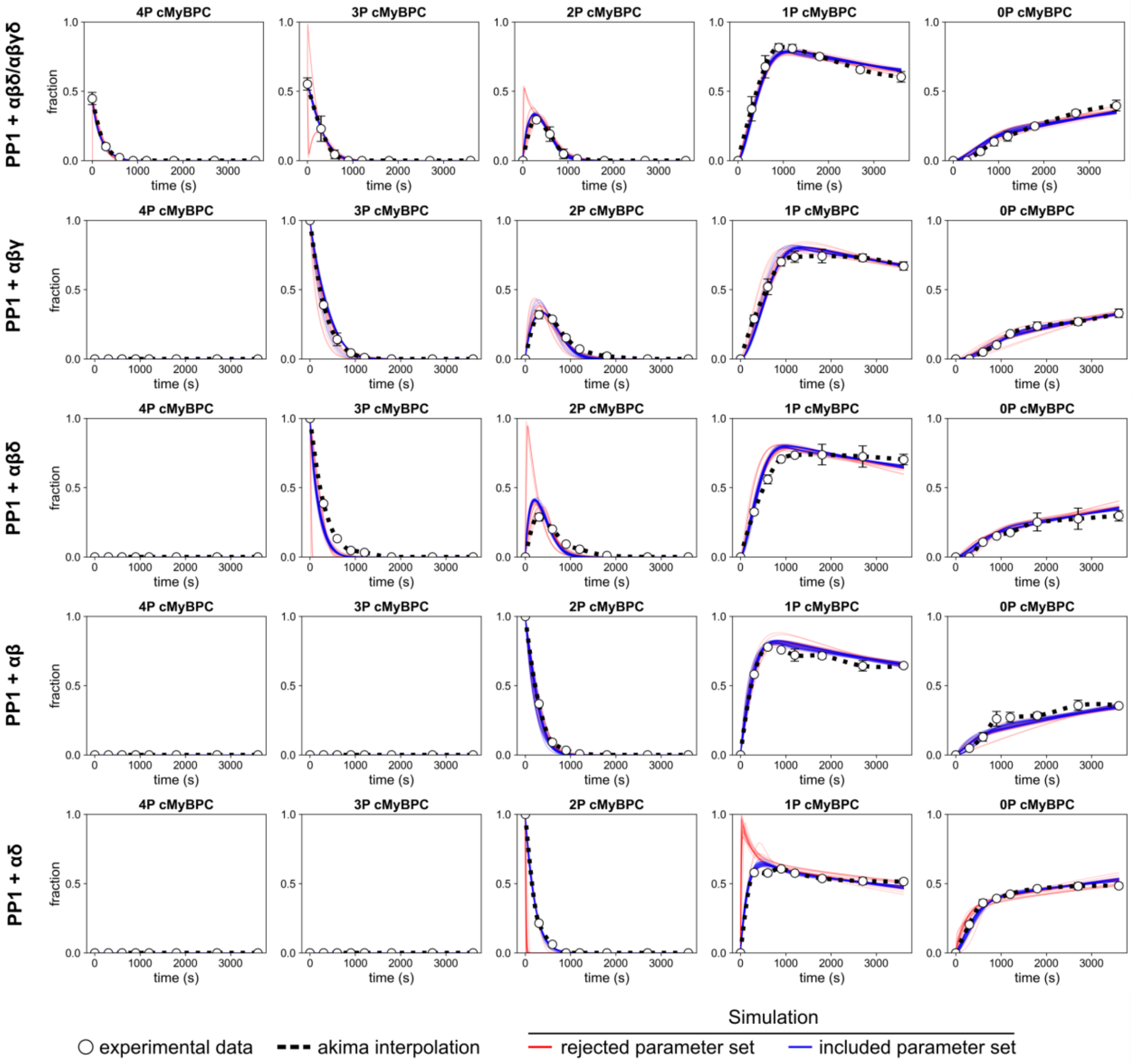

Supplementary Figure 11: Fit of model 4 (structural transition model) to PP1 time course data only (n=50 parametersets, 25 included, 25 rejected). Each experimental data point represents the mean ± SD of n = 2-3 experiments.

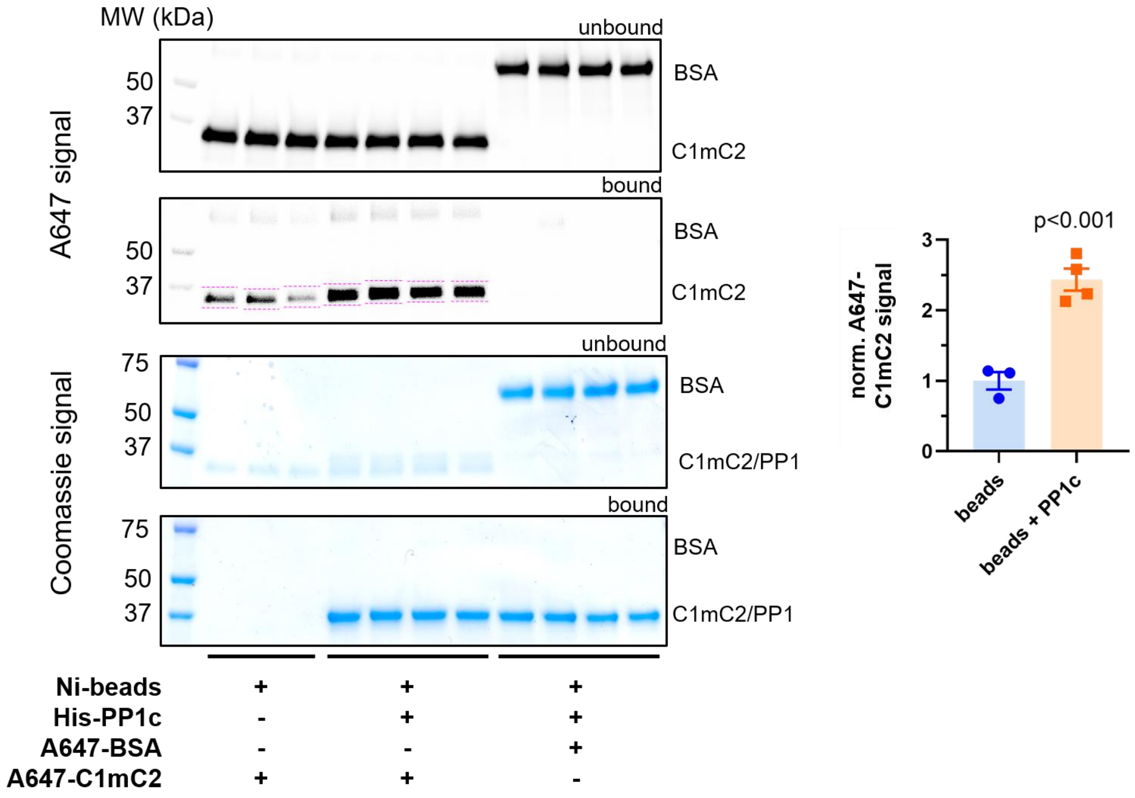

Supplementary Figure 12: Pull-down of recombinant Alexa-647-labeled C1mC2 domains by the recombinant, His_6-tagged catalytic domain of PP1. Ni-NTA beads without bait (n=3) or with His-PP1c as bait (n=4) were incubated with A647-C1mC2 or A647-BSA as a negative control (n=4). While some unspecific background binding between C1mC2 and beads was observed, PP1c-covered beads resulted in significantly higher C1mC2 retention (cf. quantification on the right). In contrast, BSA showed no binding to PP1.

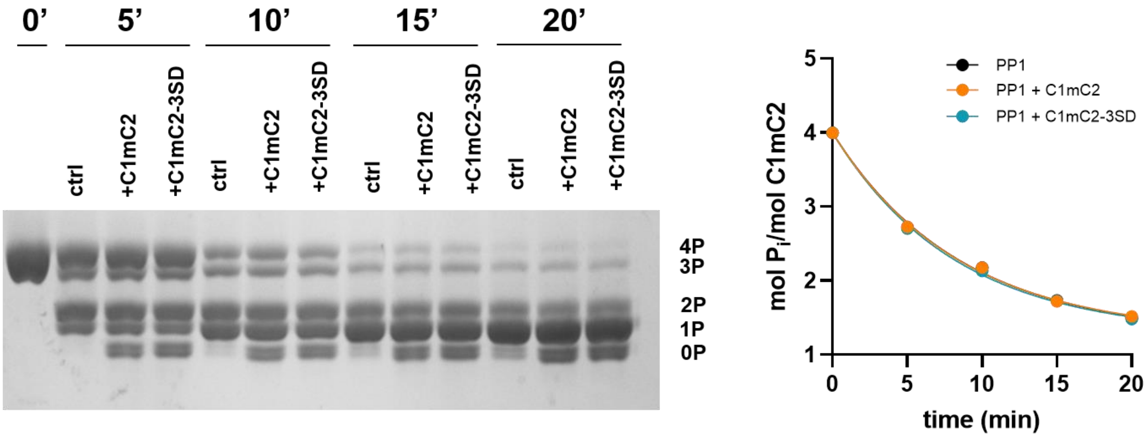

Supplementary Figure 13: Dephosphorylation time courses for fully phosphorylated C1mC2 domain by PP1 alone, PP1 preincubated with unphosphorylated C1mC2 or PP1 preincubated with phosphomimetic Ser(279/288/313)→Asp C1mC2 (n=1 for each condition). No differences in the dephosphorylation rate between groups were observed.

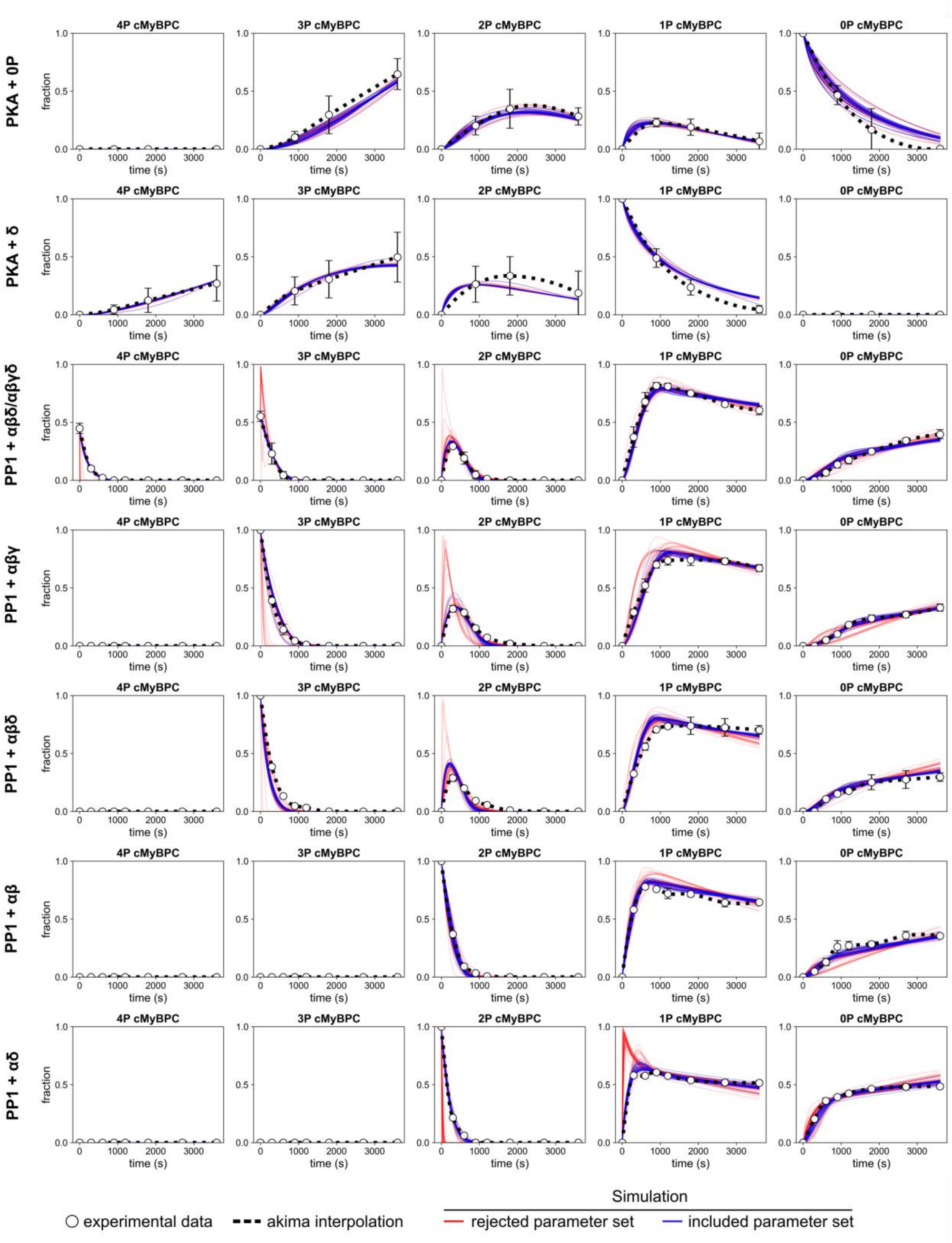

Supplementary Figure 14: Fit of model 4 (structural transition model) to all data, results shown for PKA and PP1 time course data (n=98 parametersets, 35 included, 63 rejected). Each experimental data point represents the mean ± SD of n = 2-6 experiments.

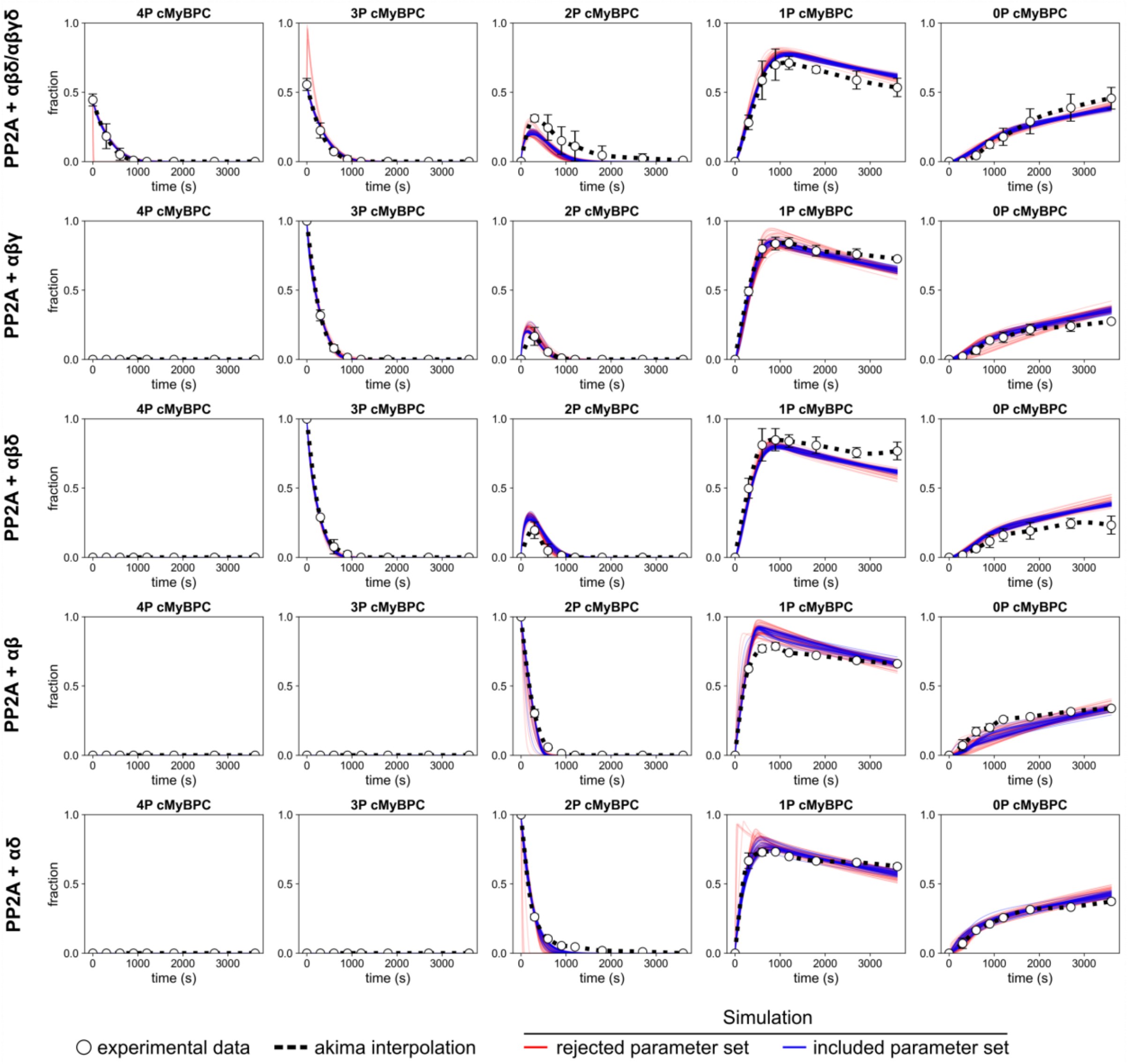

Supplementary Figure 15: Fit of model 4 (structural transition model) to all data, results shown for PP2A time course data (n=98 parametersets, 35 included, 63 rejected). Each experimental data point represents the mean ± SD of n = 2-3 experiments.

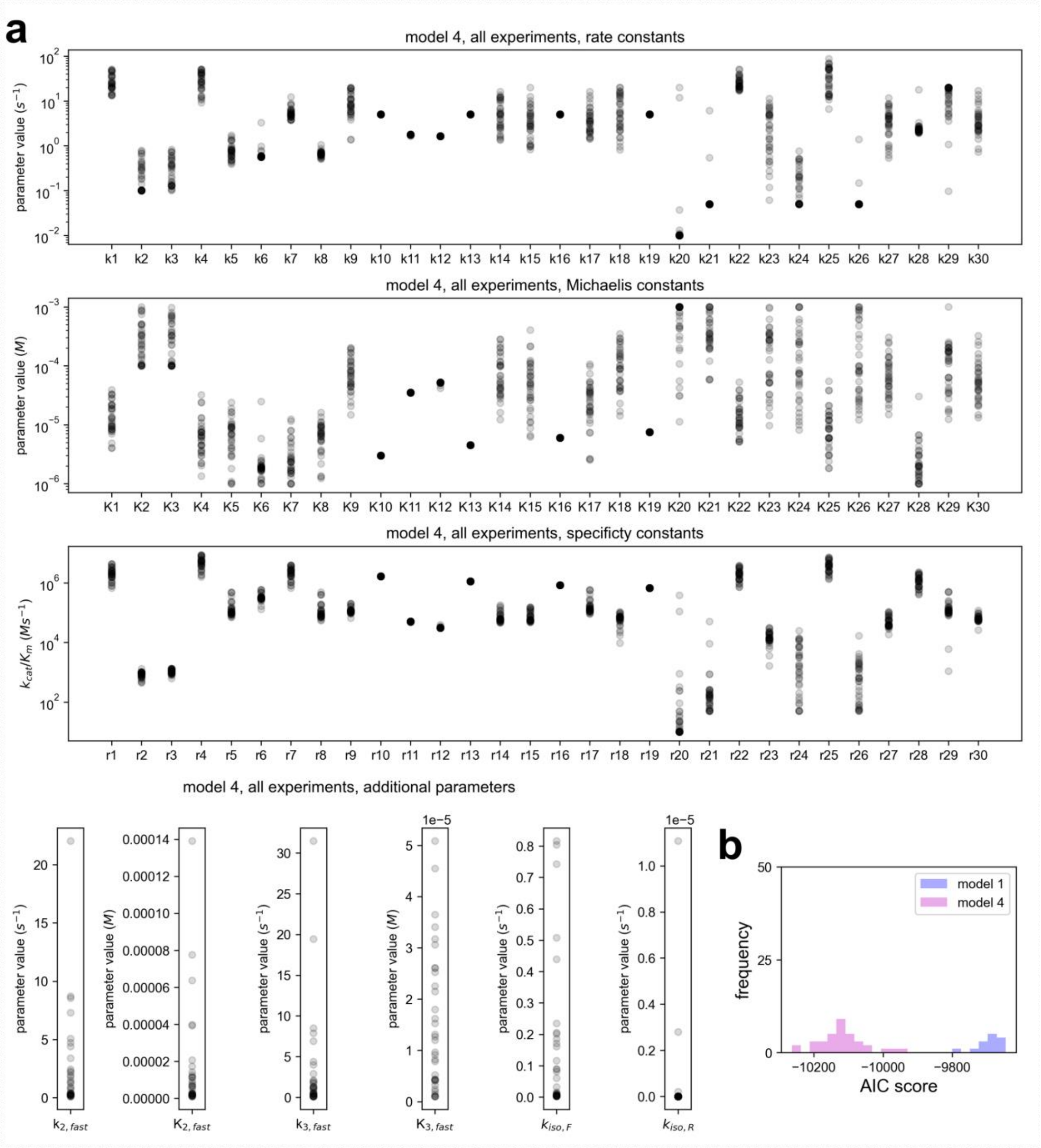

Supplementary Figure 16: **a**, Resulting parameters for model 4 after fitting the model to all datasets and filtering out poorly performing parameter sets. **b**, Comparison of model 1 and model 4 (both fitted to all datasets) using the Akaike information criterion based on included parametersets after filtering (14 parameter sets included for model 1, 35 parameter sets included for model 4). Model 4 has a significantly lower AIC score than model 1 ($p = 7.3 \times 10^{-30}$; Welch's t-test) and thus is to be preferred over model 1.

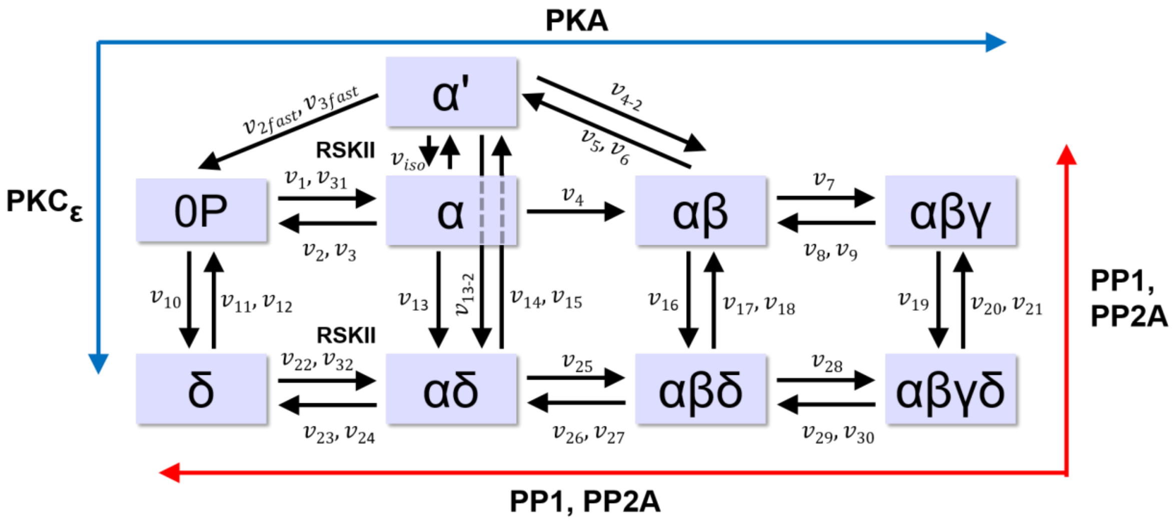

Supplementary Figure 17: Scheme of final model (model 4 + RSK2 reactions v_{31} and v_{32}).

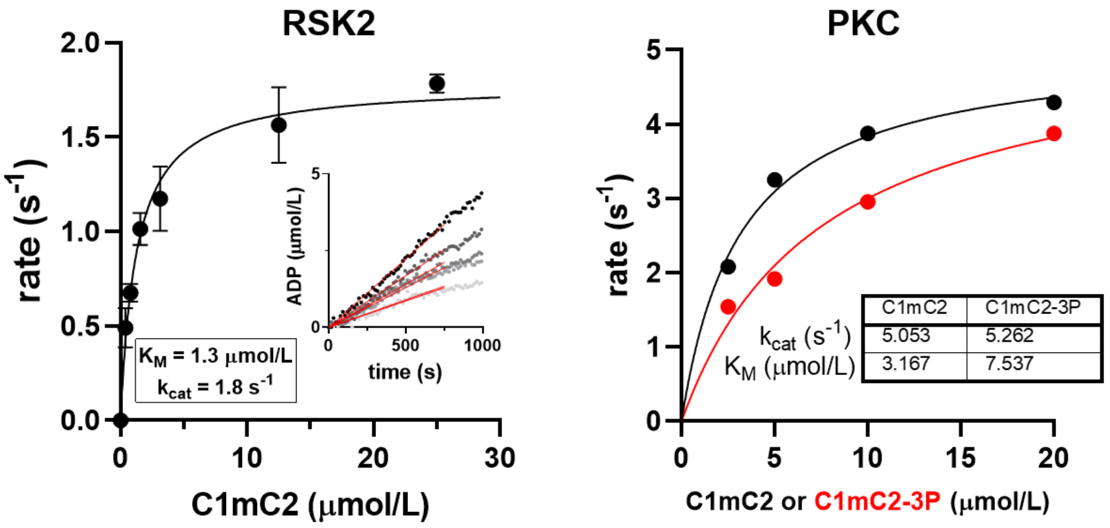

Supplementary Figure 18: Michaelis-Menten kinetics analysis for C1mC2 phosphorylation by RSK2 (left) (n=3) and PKCε (right) (n=1).

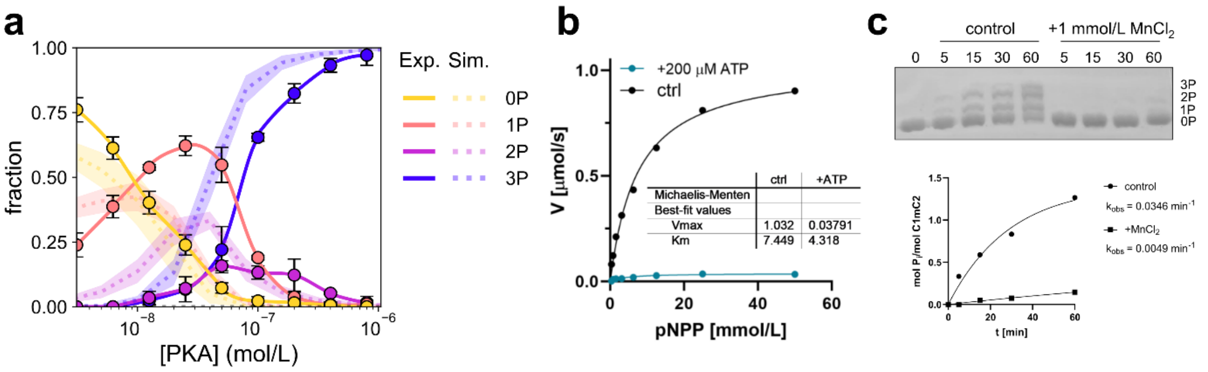

Supplementary Figure 19: a, experimental (same data as in Figure 3b of the main text) vs predicted PKA dose-response at 100 nmol/L PP1. **b**, pNPP assay in absence and presence of ATP. **c**, phosphorylation of 20 μmol/L cMyBP-C by 2000U PKA in absence and presence of $MnCl_2$.

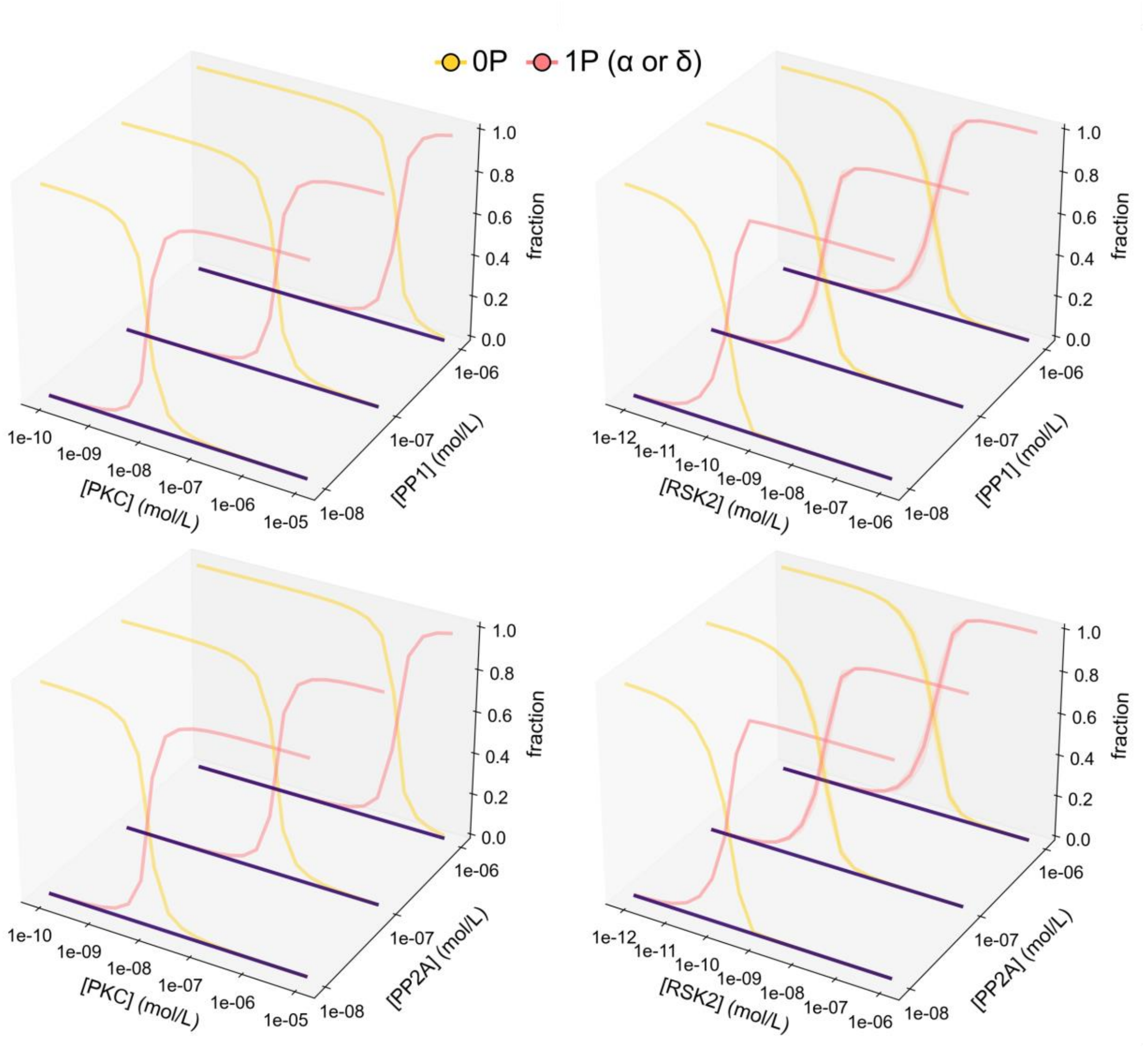

Supplementary Figure 20: Simulated steady-state phosphorylation of cMyBP-C in the presence of PP1 (top) or PP2A (bottom) and increasing concentrations of PKC (left) or RSK2 (right). Data represent mean ± SD from n=35 parameter sets.

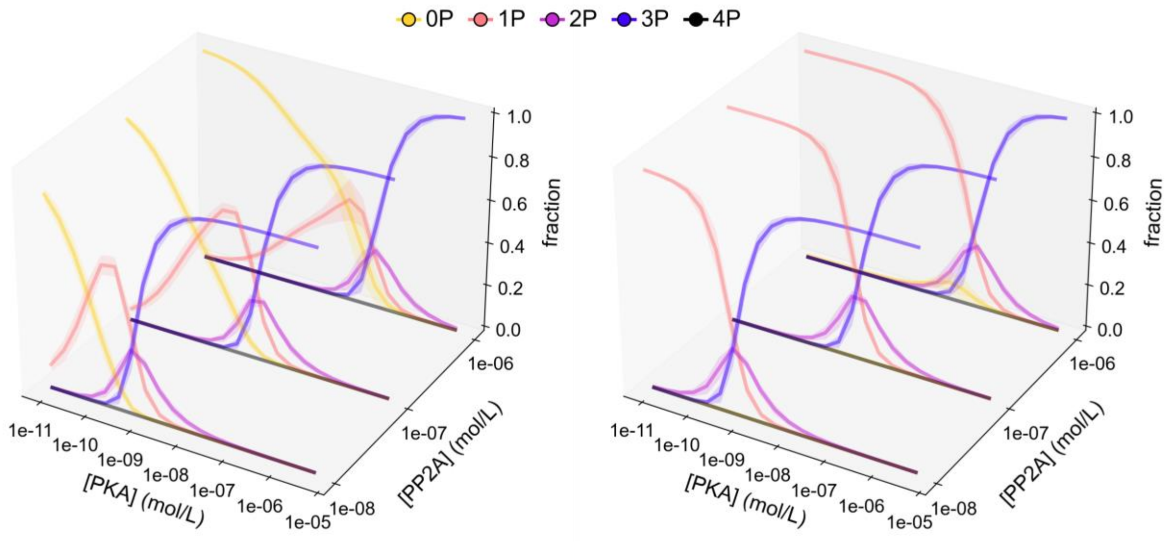

Supplementary Figure 21. Steady-state phosphorylation simulations of cMyBP-C in the presence of PP2A and increasing concentrations of PKA (left). The effect of additional 100 nmol/L RSK2 is shown in the right panel. Data represent mean ± SD from n=35 parameter sets.

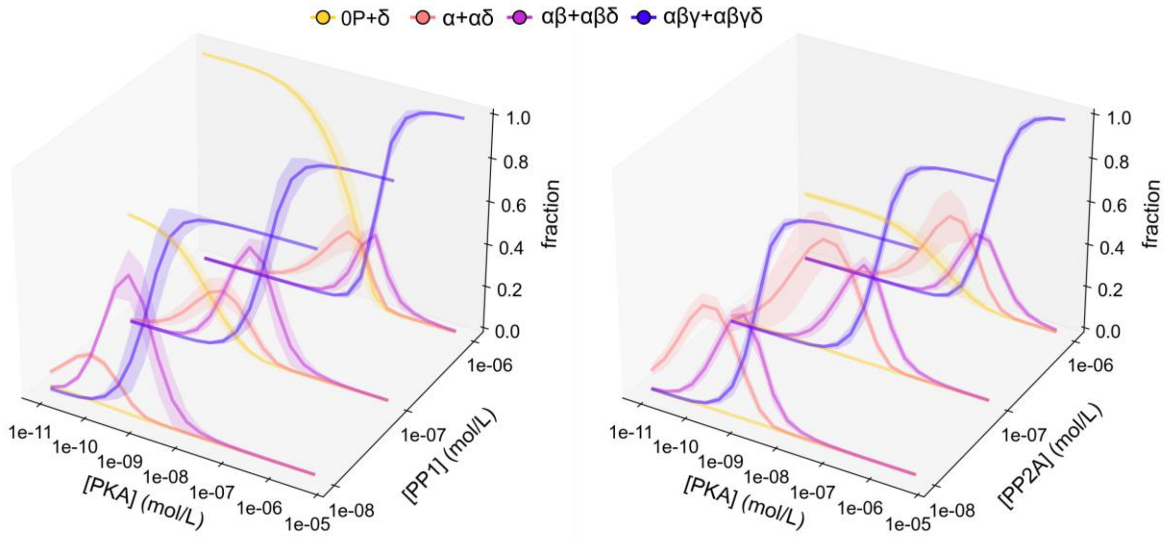

Supplementary Figure 22. Steady-state phosphorylation simulations of cMyBP-C in the presence of PKCε and PP1 (left) or PP2A (right), and increasing concentrations of PKA with species 0P+δ, α+αδ, αβ+αβδ and αβγ+αβγδ lumped together, respectively. Data represent mean ± SD from n=35 parameter sets.

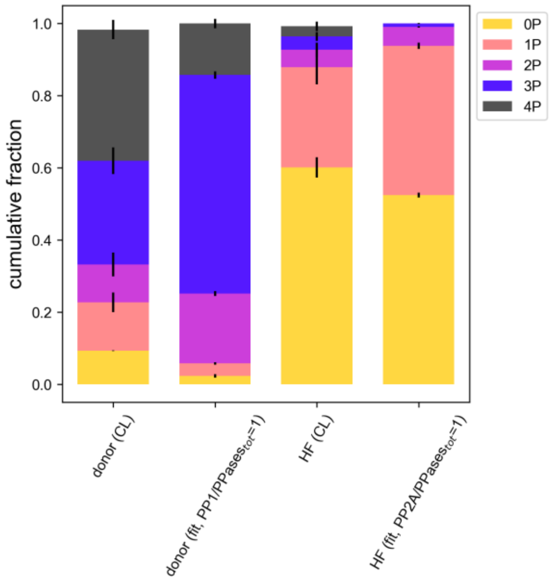

Supplementary Figure 23: Control experiment with clamped phosphatase ratios. Like in Figure 4A (main text), the model was tested for consistency with experimental data on cMyBP-C basal phosphorylation states in hearts from healthy donors or HF patients reported in Copeland *et al.* 2010 (CL) by fitting the model using only the enzyme concentrations as free parameters (fit). However, for donor heart data, the PP1/PPases$_{tot}$ was set to 1 (i.e. no PP2A was present), whereas for HF data, the PP2A/PPases$_{tot}$ was fixed to 1 (i.e. no PP1 was present). Data represent mean ± SD from n=35 parameter sets.

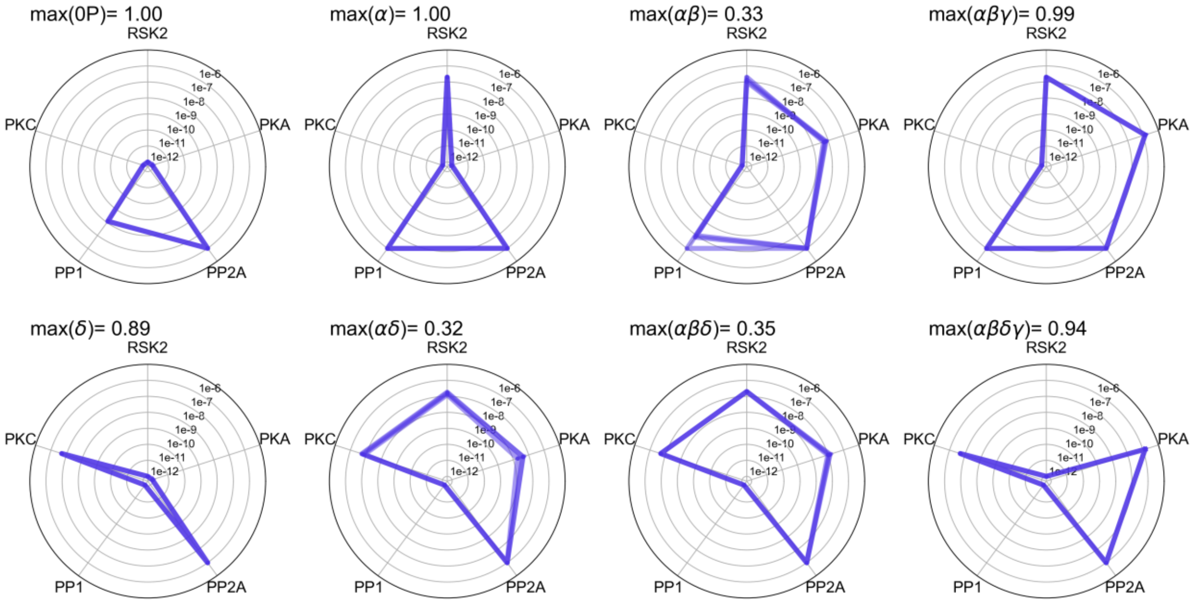

Supplementary Figure 24: Optimization of cMyBP-C states under physiological conditions. Spider plots show enzyme vectors at which the respective cMyBP-C phosphorylation is at its maximally possible fraction. Data from n=35 parameter sets.

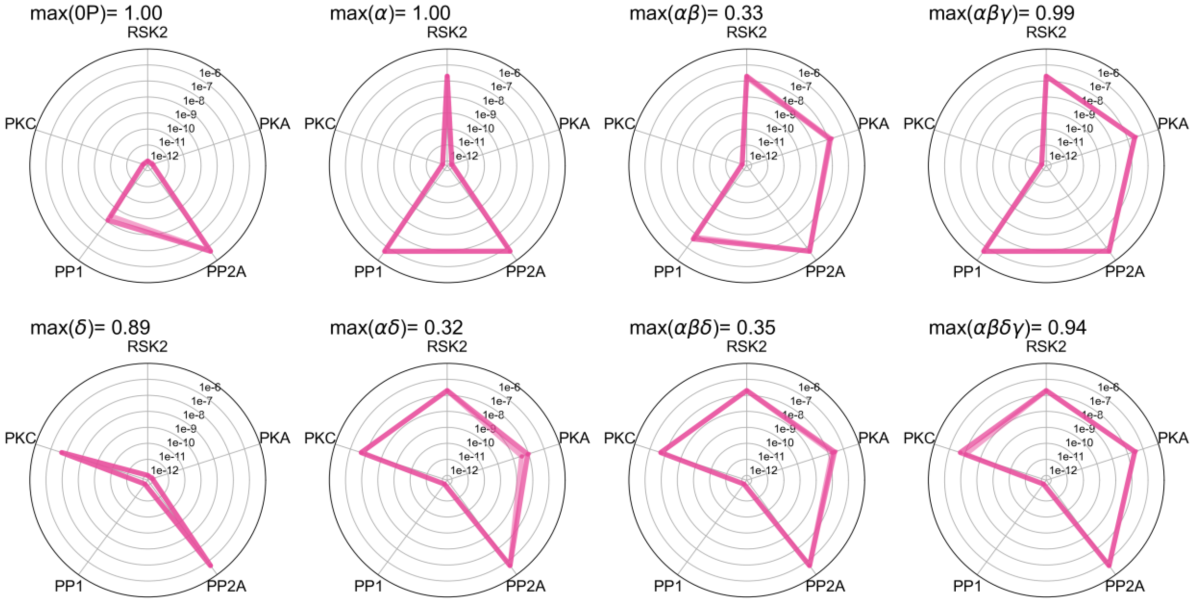

Supplementary Figure 25: Optimization of cMyBP-C states under HF conditions. Spider plots show enzyme vectors at which the respective cMyBP-C phosphorylation is at its maximally possible fraction. Data from n=35 parameter sets.

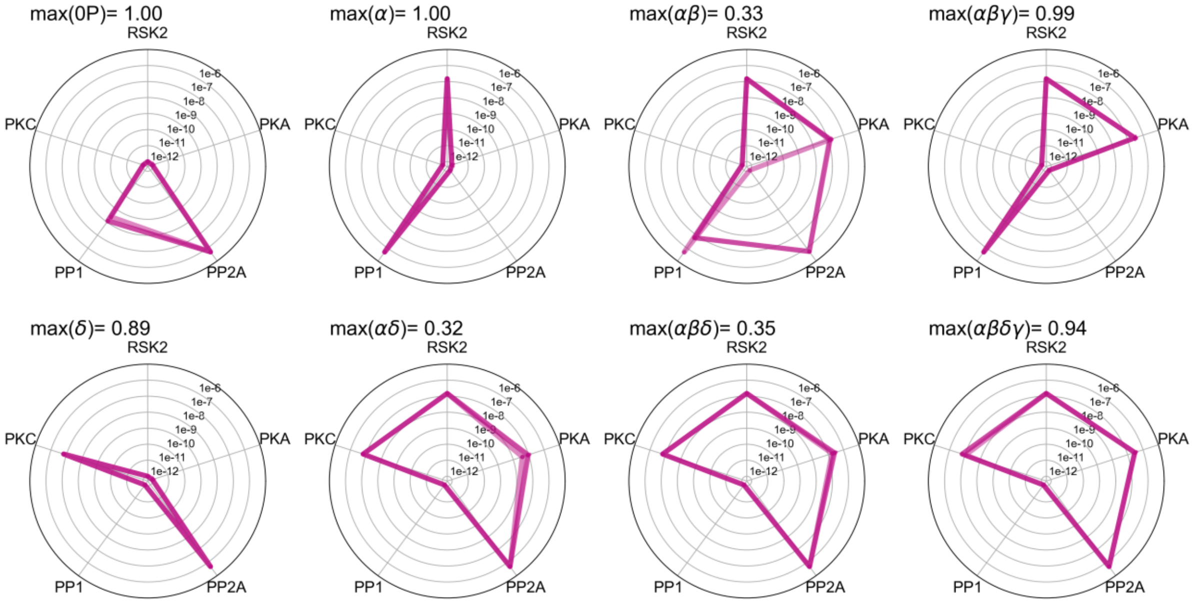

Supplementary Figure 26: Optimization of cMyBP-C states under HF conditions with additional restriction of PKC and RSK2 concentrations. Spider plots show enzyme vectors at which the respective cMyBP-C phosphorylation is at its maximally possible fraction. Data from n=35 parameter sets.

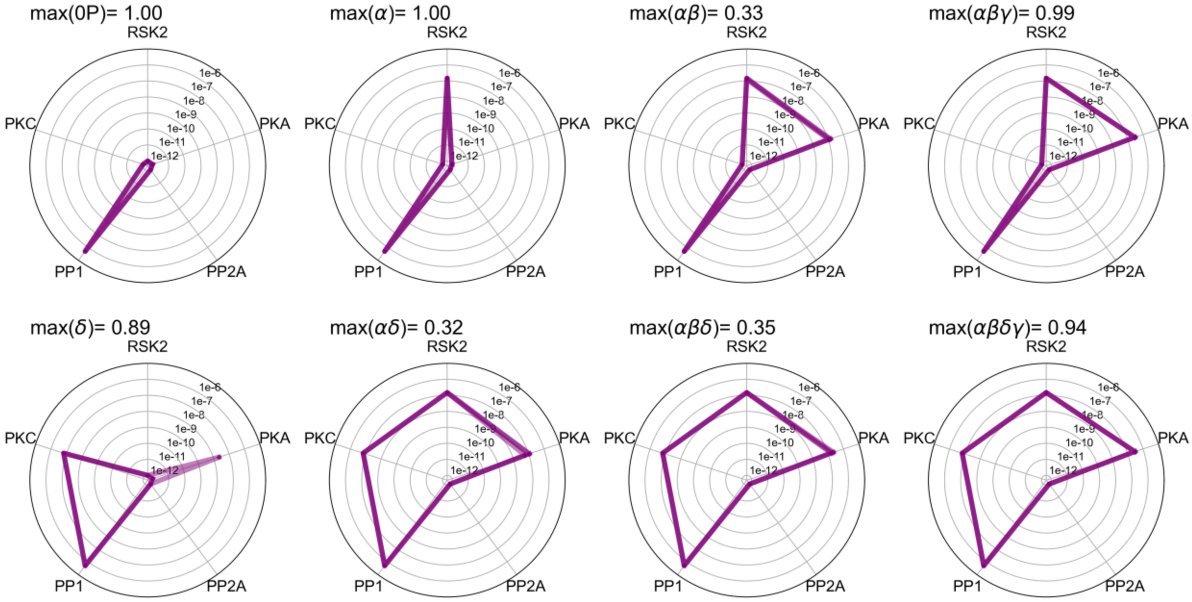

Supplementary Figure 27: Optimization of cMyBP-C states under HF conditions with additional restriction of PKC and RSK2 concentrations and clamped PP1. Spider plots show enzyme vectors at which the respective cMyBP-C phosphorylation is at its maximally possible fraction. Data from n=35 parameter sets.

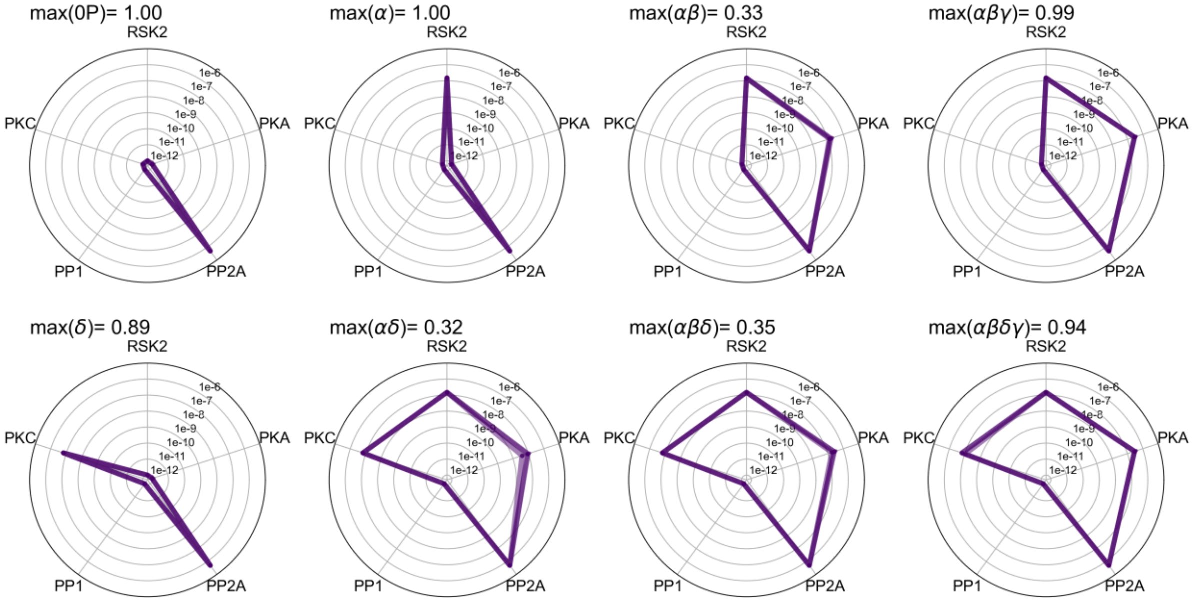

Supplementary Figure 28: Optimization of cMyBP-C states under HF conditions with additional restriction of PKC and RSK2 concentrations and clamped PP2A. Spider plots show enzyme vectors at which the respective cMyBP-C phosphorylation is at its maximally possible fraction. Data from n=35 parameter sets.

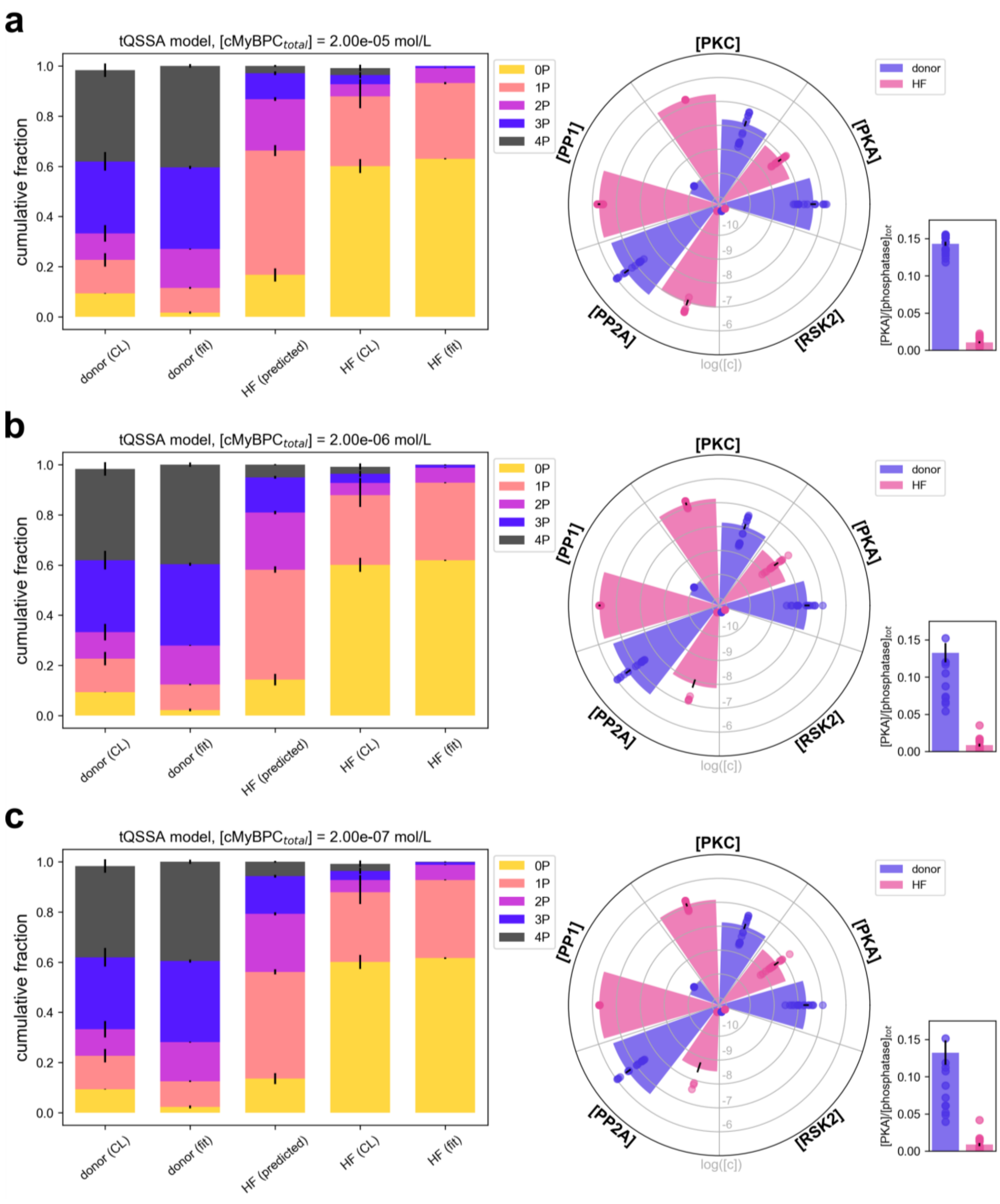

Supplementary Figure 29: cMyBP-C phosphorylation states during heart failure analyzed by tQSSA version of the model at lower substrate concentrations (**a-c**) that are comparable to enzyme concentrations. *Left*: the model was tested for consistency with experimental data on cMyBP-C basal phosphorylation states in hearts from healthy donors or HF patients reported in Copeland et al. 2010 (CL) by fitting the model using only the enzyme concentrations as free parameters (fit). Additionally, the distribution of cMyBP-C phosphorylation states during HF was predicted by starting with the enzyme concentrations fitted to the donor (CL) data followed by a decrease in [PKA] by 50% and a 2-fold increase of PP1 and PP2A concentrations consistent with previous reports on β-adrenergic receptor downregulation and phosphatase activity during HF. *Right:* comparison of the fitted enzyme concentrations underlying donor (fit) and HF (fit) data from left panel and calculated PKA/phosphatase$_{tot}$ ratios. Each fitting run has been performed with all of the n=18 parameter sets. Values represent mean ± SEM.

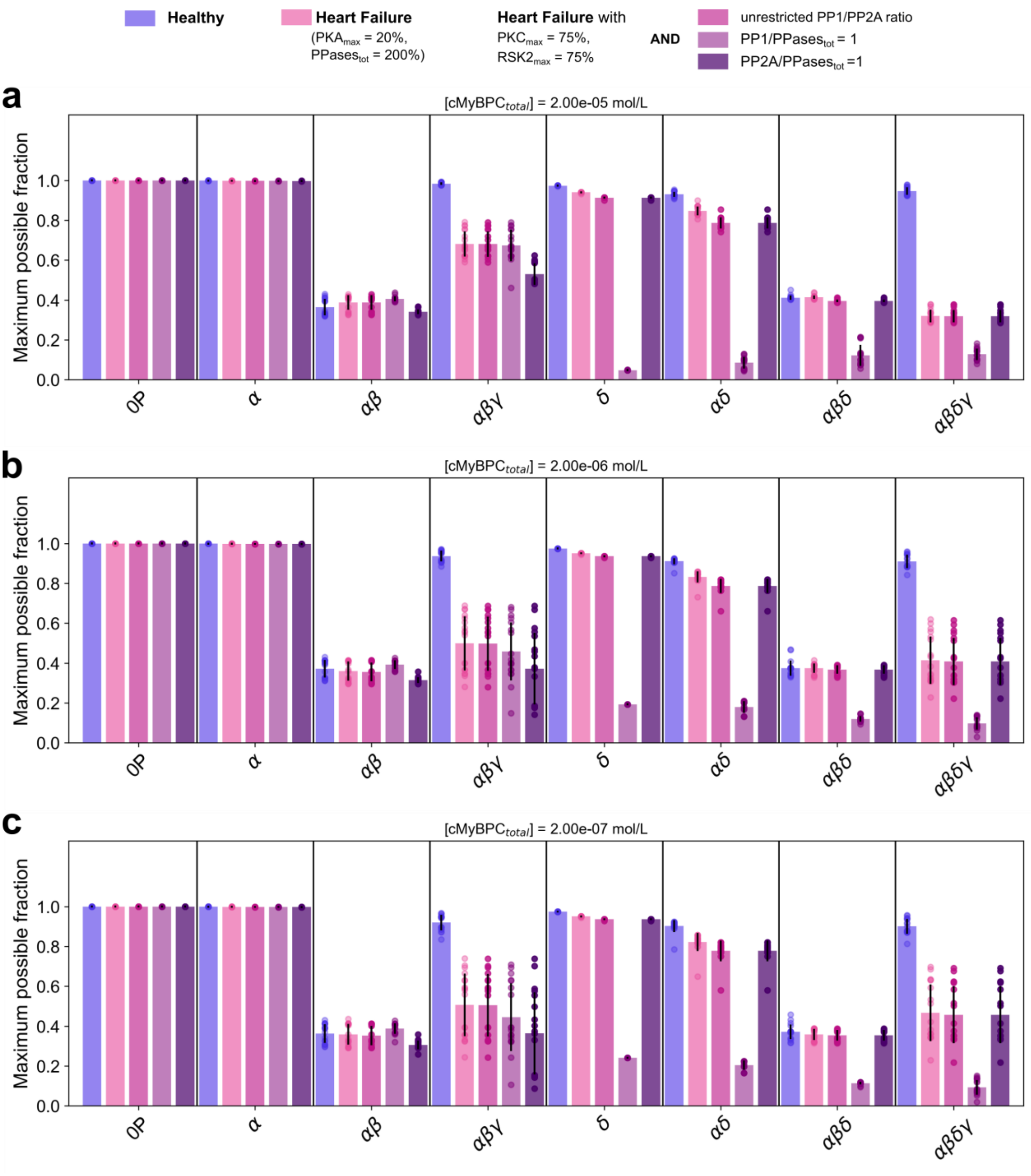

Supplementary Figure 30 Maximally achievable fraction for each cMyBP-C phosphorylation state under various conditions as analyzed by tQSSA version of the model at lower substrate concentrations (**a-c**) that are comparable to enzyme concentrations. For each phosphorylation state, algorithmic optimization was used to find the enzyme vector [PKA, PKC, RSK2, PP1, PP2A] (within a physiological range) that maximizes the phosphorylation state under consideration. To probe the effect of perturbing other enzymes during HF, PKC and RSK2 concentrations were further restricted and PP1/PP2A ratio was either allowed to vary freely (crimson), or phosphatases were fixed to PP2A/PPasetot = 1 (purple) or PP1/PPasetot = 1 (dark purple). Each optimization run has been performed with all of the n=18 parameter sets. Values represent mean ± SEM.

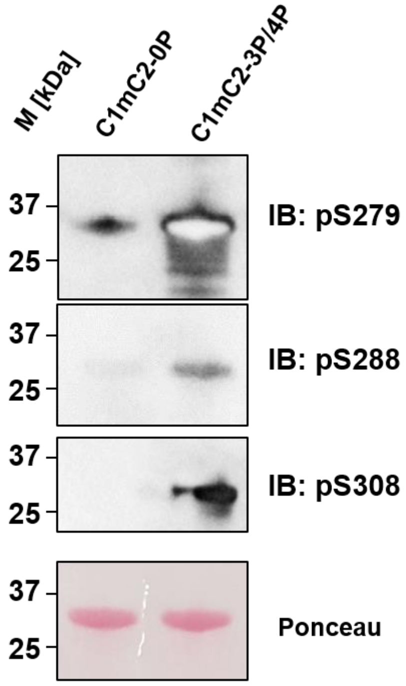

Supplementary Figure 31 Antibody validation. Unphosphorylated and almost fully phosphorylated C1mC2 fragments (containing a mixture of pS279,pS288,pS308- and pS279,pS288,pS308,pS313-C1mC2) were probed with the indicated site-specific antibodies.

Supplementary Tables

Supplementary Table 1: Statistical comparisons of Hill-coefficients n_H and EC50 values

Dose response simulations	Comparisons	Test	p-value	Significant?
PKA vs PP1	n_H at different [Phosphatase] (10^{-8} vs 10^{-7} vs 10^{-6} mol/L)	Kruskal-Wallis	$7.62x10^{-12}$	Yes
PKA + 100 nmol/L RSK2 vs PP1	n_H at different [Phosphatase] (10^{-8} vs 10^{-7} vs 10^{-6} mol/L)	Kruskal-Wallis	$2.91x10^{-10}$	Yes
PKA + 100 nmol/L PKC vs PP1	n_H at different [Phosphatase] (10^{-8} vs 10^{-7} vs 10^{-6} mol/L)	Kruskal-Wallis	$1.02x10^{-18}$	Yes
PKA vs PP2A	n_H at different [Phosphatase] (10^{-8} vs 10^{-7} vs 10^{-6} mol/L)	Kruskal-Wallis	$2.53x10^{-8}$	Yes
PKA + 100 nmol/L RSK2 vs PP2A	n_H at different [Phosphatase] (10^{-8} vs 10^{-7} vs 10^{-6} mol/L)	Kruskal-Wallis	$6.93x10^{-6}$	Yes
PKA + 100 nmol/L PKC vs PP2A	n_H at different [Phosphatase] (10^{-8} vs 10^{-7} vs 10^{-6} mol/L)	Kruskal-Wallis	$4.80x10^{-14}$	Yes
PKA vs PP1,<br>PKA + 100 nmol/L RSK2 vs PP1,<br>PKA + 100 nmol/L PKC vs PP1	n_H across dose responses at [Phosphatase] = 10^{-8} mol/L	Kruskal-Wallis	$1.52x10^{-14}$	Yes
PKA vs PP1,<br>PKA + 100 nmol/L RSK2 vs PP1,<br>PKA + 100 nmol/L PKC vs PP1	n_H across dose responses at [Phosphatase] = 10^{-7} mol/L	Kruskal-Wallis	$5.23x10^{-15}$	Yes
PKA vs PP1,<br>PKA + 100 nmol/L RSK2 vs PP1,<br>PKA + 100 nmol/L PKC vs PP1	n_H across dose responses at [Phosphatase] = 10^{-6} mol/L	Kruskal-Wallis	0.07	No
PKA vs PP2A,<br>PKA + 100 nmol/L RSK2 vs PP2A,<br>PKA + 100 nmol/L PKC vs PP2A	n_H across dose responses at [Phosphatase] = 10^{-8} mol/L	Kruskal-Wallis	$6.25x10^{-7}$	Yes
PKA vs PP2A,<br>PKA + 100 nmol/L RSK2 vs PP2A,<br>PKA + 100 nmol/L PKC vs PP2A	n_H across dose responses at [Phosphatase] = 10^{-7} mol/L	Kruskal-Wallis	0.065	No
PKA vs PP2A,<br>PKA + 100 nmol/L RSK2 vs PP2A,<br>PKA + 100 nmol/L PKC vs PP2A	n_H across dose responses at [Phosphatase] = 10^{-6} mol/L	Kruskal-Wallis	0.0016	Yes
PKA vs PP1, PKA vs PP2A	n_H for PP1 vs PP2A at [Phosphatase] = 10^{-8} mol/L	Mann-Whitney	$8.21x10^{-6}$	Yes
PKA vs PP1, PKA vs PP2A	n_H for PP1 vs PP2A at [Phosphatase] = 10^{-7} mol/L	Mann-Whitney	$2.51x10^{-9}$	Yes
PKA vs PP1, PKA vs PP2A	n_H for PP1 vs PP2A at [Phosphatase] = 10^{-6} mol/L	Mann-Whitney	$4.70x10^{-9}$	Yes
PKA + 100 nmol/L RSK2 vs PP1,<br>PKA + 100 nmol/L RSK2 vs PP2A	n_H for PP1 vs PP2A at [Phosphatase] = 10^{-8} mol/L	Mann-Whitney	$1.29x10^{-5}$	Yes
PKA + 100 nmol/L RSK2 vs PP1,<br>PKA + 100 nmol/L RSK2 vs PP2A	n_H for PP1 vs PP2A at [Phosphatase] = 10^{-7} mol/L	Mann-Whitney	$5.06x10^{-9}$	Yes
PKA + 100 nmol/L RSK2 vs PP1,<br>PKA + 100 nmol/L RSK2 vs PP2A	n_H for PP1 vs PP2A at [Phosphatase] = 10^{-6} mol/L	Mann-Whitney	$1.45x10^{-8}$	Yes
PKA + 100 nmol/L PKC vs PP1,<br>PKA + 100 nmol/L PKC vs PP2A	n_H for PP1 vs PP2A at [Phosphatase] = 10^{-8} mol/L	Mann-Whitney	0.89	No
PKA + 100 nmol/L PKC vs PP1,<br>PKA + 100 nmol/L PKC vs PP2A	n_H for PP1 vs PP2A at [Phosphatase] = 10^{-7} mol/L	Mann-Whitney	0.5	No
PKA + 100 nmol/L PKC vs PP1,<br>PKA + 100 nmol/L PKC vs PP2A	n_H for PP1 vs PP2A at [Phosphatase] = 10^{-6} mol/L	Mann-Whitney	$3.97x10^{-10}$	Yes
PKA vs PP1, PKA vs PP2A	EC50 for PP1 vs PP2A at [Phosphatase] = 10^{-8} mol/L	Mann-Whitney	$6.51x10^{-5}$	Yes
PKA vs PP1, PKA vs PP2A	EC50 for PP1 vs PP2A at [Phosphatase] = 10^{-7} mol/L	Mann-Whitney	$1.37x10^{-8}$	Yes
PKA vs PP1, PKA vs PP2A	EC50 for PP1 vs PP2A at [Phosphatase] = 10^{-6} mol/L	Mann-Whitney	$1.37x10^{-8}$	Yes
PKA + 100 nmol/L RSK2 vs PP1,<br>PKA + 100 nmol/L RSK2 vs PP2A	EC50 for PP1 vs PP2A at [Phosphatase] = 10^{-8} mol/L	Mann-Whitney	$5.32x10^{-7}$	Yes
PKA + 100 nmol/L RSK2 vs PP1,<br>PKA + 100 nmol/L RSK2 vs PP2A	EC50 for PP1 vs PP2A at [Phosphatase] = 10^{-7} mol/L	Mann-Whitney	$2.05x10^{-8}$	Yes
PKA + 100 nmol/L RSK2 vs PP1,<br>PKA + 100 nmol/L RSK2 vs PP2A	EC50 for PP1 vs PP2A at [Phosphatase] = 10^{-6} mol/L	Mann-Whitney	$1.37x10^{-8}$	Yes
PKA + 100 nmol/L PKC vs PP1,<br>PKA + 100 nmol/L PKC vs PP2A	EC50 for PP1 vs PP2A at [Phosphatase] = 10^{-8} mol/L	Mann-Whitney	$6.00x10^{-5}$	Yes
PKA + 100 nmol/L PKC vs PP1,<br>PKA + 100 nmol/L PKC vs PP2A	EC50 for PP1 vs PP2A at [Phosphatase] = 10^{-7} mol/L	Mann-Whitney	$8.61x10^{-8}$	Yes
PKA + 100 nmol/L PKC vs PP1,<br>PKA + 100 nmol/L PKC vs PP2A	EC50 for PP1 vs PP2A at [Phosphatase] = 10^{-6} mol/L	Mann-Whitney	$6.74x10^{-11}$	Yes

Supplementary Notes

Model equations

Model 1 (Michaelis-Menten-type reactions only)

Substrate competition terms

$$\kappa_{PKA} = \frac{0P}{K_1} + \frac{\alpha}{K_4} + \frac{\alpha\beta}{K_7} + \frac{\delta}{K_{22}} + \frac{\alpha\delta}{K_{25}} + \frac{\alpha\beta\delta}{K_{28}}$$

$$\kappa_{PKC} = \frac{0P}{K_{10}} + \frac{\alpha}{K_{13}} + \frac{\alpha\beta}{K_{16}} + \frac{\alpha\beta\gamma}{K_{19}}$$

$$\kappa_{PP1} = \frac{\alpha}{K_2} + \frac{\alpha\beta}{K_5} + \frac{\alpha\beta\gamma}{K_8} + \frac{\delta}{K_{11}} + \frac{\alpha\delta}{K_{14}} + \frac{\alpha\delta}{K_{23}} + \frac{\alpha\beta\delta}{K_{17}} + \frac{\alpha\beta\delta}{K_{26}} + \frac{\alpha\beta\gamma\delta}{K_{20}} + \frac{\alpha\beta\gamma\delta}{K_{29}}$$

$$\kappa_{PP2A} = \frac{\alpha}{K_3} + \frac{\alpha\beta}{K_6} + \frac{\alpha\beta\gamma}{K_9} + \frac{\delta}{K_{12}} + \frac{\alpha\delta}{K_{15}} + \frac{\alpha\delta}{K_{24}} + \frac{\alpha\beta\delta}{K_{18}} + \frac{\alpha\beta\delta}{K_{27}} + \frac{\alpha\beta\gamma\delta}{K_{21}} + \frac{\alpha\beta\gamma\delta}{K_{30}}$$

Rate laws

PKA

$$v_1 = \frac{k_1 \cdot PKA \cdot 0P}{K_1 \cdot \left(1 + \kappa_{PKA} - \frac{0P}{K_1}\right) + 0P}$$

$$v_4 = \frac{k_4 \cdot PKA \cdot \alpha}{K_4 \cdot \left(1 + \kappa_{PKA} - \frac{\alpha}{K_4}\right) + \alpha}$$

$$v_7 = \frac{k_7 \cdot PKA \cdot \alpha\beta}{K_7 \cdot \left(1 + \kappa_{PKA} - \frac{\alpha\beta}{K_7}\right) + \alpha\beta}$$

$$v_{22} = \frac{k_{22} \cdot PKA \cdot \delta}{K_{22} \cdot \left(1 + \kappa_{PKA} - \frac{\delta}{K_{22}}\right) + \delta}$$

$$v_{25} = \frac{k_{25} \cdot PKA \cdot \alpha\delta}{K_{25} \cdot \left(1 + \kappa_{PKA} - \frac{\alpha\delta}{K_{25}}\right) + \alpha\delta}$$

$$v_{28} = \frac{k_{28} \cdot PKA \cdot \alpha\beta\delta}{K_{28} \cdot \left(1 + \kappa_{PKA} - \frac{\alpha\beta\delta}{K_{28}}\right) + \alpha\beta\delta}$$

PKC

$$v_{10} = \frac{k_{10} \cdot PKC \cdot 0P}{K_{10} \cdot \left(1 + \kappa_{PKC} - \frac{0P}{K_{10}}\right) + 0P}$$

$$v_{13} = \frac{k_{13} \cdot PKC \cdot \alpha}{K_{13} \cdot \left(1 + \kappa_{PKC} - \frac{\alpha}{K_{13}}\right) + \alpha}$$

$$v_{16} = \frac{k_{16} \cdot PKC \cdot \alpha\beta}{K_{16} \cdot \left(1 + \kappa_{PKC} - \frac{\alpha\beta}{K_{16}}\right) + \alpha\beta}$$

$$v_{19} = \frac{k_{19} \cdot PKC \cdot \alpha\beta\gamma}{K_{19} \cdot \left(1 + \kappa_{PKC} - \frac{\alpha\beta\gamma}{K_{19}}\right) + \alpha\beta\gamma}$$

PP1

$$v_2 = \frac{k_2 \cdot PP1 \cdot \alpha}{K_2 \cdot \left(1 + \kappa_{PP1} - \frac{\alpha}{K_2}\right) + \alpha}$$

$$v_5 = \frac{k_5 \cdot PP1 \cdot \alpha\beta}{K_5 \cdot \left(1 + \kappa_{PP1} - \frac{\alpha\beta}{K_5}\right) + \alpha\beta}$$

$$v_8 = \frac{k_8 \cdot PP1 \cdot \alpha\beta\gamma}{K_8 \cdot \left(1 + \kappa_{PP1} - \frac{\alpha\beta\gamma}{K_8}\right) + \alpha\beta\gamma}$$

$$v_{11} = \frac{k_{11} \cdot PP1 \cdot \delta}{K_{11} \cdot \left(1 + \kappa_{PP1} - \frac{\delta}{K_{11}}\right) + \delta}$$

$$v_{14} = \frac{k_{14} \cdot PP1 \cdot \alpha\delta}{K_{14} \cdot \left(1 + \kappa_{PP1} - \frac{\alpha\delta}{K_{14}}\right) + \alpha\delta}$$

$$v_{17} = \frac{k_{17} \cdot PP1 \cdot \alpha\beta\delta}{K_{17} \cdot \left(1 + \kappa_{PP1} - \frac{\alpha\beta\delta}{K_{17}}\right) + \alpha\beta\delta}$$

$$v_{20} = \frac{k_{20} \cdot PP1 \cdot \alpha\beta\gamma\delta}{K_{20} \cdot \left(1 + \kappa_{PP1} - \frac{\alpha\beta\gamma\delta}{K_{20}}\right) + \alpha\beta\gamma\delta}$$

$$v_{23} = \frac{k_{23} \cdot PP1 \cdot \alpha\delta}{K_{23} \cdot \left(1 + \kappa_{PP1} - \frac{\alpha\delta}{K_{23}}\right) + \alpha\delta}$$

$$v_{26} = \frac{k_{26} \cdot PP1 \cdot \alpha\beta\delta}{K_{26} \cdot \left(1 + \kappa_{PP1} - \frac{\alpha\beta\delta}{K_{26}}\right) + \alpha\beta\delta}$$

$$v_{29} = \frac{k_{29} \cdot PP1 \cdot \alpha\beta\gamma\delta}{K_{29} \cdot \left(1 + \kappa_{PP1} - \frac{\alpha\beta\gamma\delta}{K_{29}}\right) + \alpha\beta\gamma\delta}$$

PP2A

$$v_3 = \frac{k_3 \cdot PP2A \cdot \alpha}{K_3 \cdot \left(1 + \kappa_{PP2A} - \frac{\alpha}{K_3}\right) + \alpha}$$

$$v_6 = \frac{k_6 \cdot PP2A \cdot \alpha\beta}{K_6 \cdot \left(1 + \kappa_{PP2A} - \frac{\alpha\beta}{K_6}\right) + \alpha\beta}$$

$$v_9 = \frac{k_9 \cdot PP2A \cdot \alpha\beta\gamma}{K_9 \cdot \left(1 + \kappa_{PP2A} - \frac{\alpha\beta\gamma}{K_0}\right) + \alpha\beta\gamma}$$

$$v_{12} = \frac{k_{12} \cdot PP2A \cdot \delta}{K_{12} \cdot \left(1 + \kappa_{PP2A} - \frac{\delta}{K_{12}}\right) + \delta}$$

$$v_{15} = \frac{k_{15} \cdot PP2A \cdot \alpha\delta}{K_{15} \cdot \left(1 + \kappa_{PP2A} - \frac{\alpha\delta}{K_{15}}\right) + \alpha\delta}$$

$$v_{18} = \frac{k_{18} \cdot PP2A \cdot \alpha\beta\delta}{K_{18} \cdot \left(1 + \kappa_{PP2A} - \frac{\alpha\beta\delta}{K_{18}}\right) + \alpha\beta\delta}$$

$$v_{21} = \frac{k_{21} \cdot PP2A \cdot \alpha\beta\gamma\delta}{K_{21} \cdot \left(1 + \kappa_{PP2A} - \frac{\alpha\beta\gamma\delta}{K_{21}}\right) + \alpha\beta\gamma\delta}$$

$$v_{24} = \frac{k_{24} \cdot PP2A \cdot \alpha\delta}{K_{24} \cdot \left(1 + \kappa_{PP2A} - \frac{\alpha\delta}{K_{24}}\right) + \alpha\delta}$$

$$v_{27} = \frac{k_{27} \cdot PP2A \cdot \alpha\beta\delta}{K_{27} \cdot \left(1 + \kappa_{PP2A} - \frac{\alpha\beta\delta}{K_{27}}\right) + \alpha\beta\delta}$$

$$v_{30} = \frac{k_{30} \cdot PP2A \cdot \alpha\beta\gamma\delta}{K_{30} \cdot \left(1 + \kappa_{PP2A} - \frac{\alpha\beta\gamma\delta}{K_{30}}\right) + \alpha\beta\gamma\delta}$$

ODEs

$$\frac{d}{dt}0P = v_2 + v_3 + v_{11} + v_{12} - v_1 - v_{10}$$

$$\frac{d}{dt}\alpha = v_1 + v_5 + v_6 + v_{14} + v_{15} - v_2 - v_3 - v_4 - v_{13}$$

$$\frac{d}{dt}\alpha\beta = v_4 + v_8 + v_9 + v_{17} + v_{18} - v_5 - v_6 - v_7 - v_{16}$$

$$\frac{d}{dt}\alpha\beta\gamma = v_7 + v_{20} + v_{21} - v_8 - v_9 - v_{19}$$

$$\frac{d}{dt}\delta = v_{10} + v_{23} + v_{24} - v_{11} - v_{12} - v_{22}$$

$$\frac{d}{dt}\alpha\delta = v_{13} + v_{22} + v_{26} + v_{27} - v_{14} - v_{15} - v_{23} - v_{24} - v_{25}$$

$$\frac{d}{dt}\alpha\beta\delta = v_{16} + v_{25} + v_{29} + v_{30} - v_{17} - v_{18} - v_{26} - v_{27} - v_{28}$$

$$\frac{d}{dt}\alpha\beta\gamma\delta = v_{19} + v_{28} - v_{20} - v_{21} - v_{29} - v_{30}$$

Model 2 (Phenomenological model of increased α-dephosphorylation in presence of 2P/3P cMyBP-C)

All competition terms, rate laws and ODEs are identical to model 1, except for rate law v_2, which was multiplied with a 2P/3P-cMyB-C dependent activation term:

$v_2 = \left(\frac{k_2 \cdot PP1 \cdot \alpha}{K_2 \cdot \left(1 + \kappa_{PP1} - \frac{\alpha}{K_2}\right) + \alpha}\right) \cdot (1 + f_{act} \cdot h)$, where the parameter $f_{act} \geq 0$ denotes a maximum activation factor and $h = \frac{r_{2P/3P}}{K_{act} + r_{2P/3P}}$ is a hyperbolic function of the relative amount of bis- and trisphosphorylated cMyBP-C $r_{2P/3P} = \frac{\alpha\beta + \alpha\delta + \alpha\beta\gamma + \alpha\beta\delta}{0P + \alpha + \alpha\beta + \alpha\beta\gamma + \delta + \alpha\delta + \alpha\beta\delta + \alpha\beta\gamma\delta}$, with a half-saturation constant K_{act}.

Model 3 (Allosteric activation of α-dephosphorylation by 2P cMyBP-C)

All competition terms, rate laws and ODEs are identical to model 1, except for rate law v_2, which in model 3 accounts for allosteric activation by 2P:

$$v_2 = \frac{k_2 \cdot PP1 \cdot \alpha + k_A \cdot PP1 \cdot \alpha \cdot \frac{\alpha\beta + \alpha\delta}{\lambda K_A}}{K_2 + \frac{K_2 \cdot (\alpha\beta + \alpha\delta)}{K_A} + \frac{\alpha \cdot (\alpha\beta + \alpha\delta)}{\lambda K_A} + K_2 \cdot \left(\kappa_{PP1} - \frac{\alpha}{K_2}\right) + \alpha},$$

where λ, k_A, K_A are parameters (see methods for details).

Model 4

Substrate competition terms

$$\kappa_{PKA} = \frac{0P}{K_1} + \frac{\alpha+\alpha'}{K_4} + \frac{\alpha\beta}{K_7} + \frac{\delta}{K_{22}} + \frac{\alpha\delta}{K_{25}} + \frac{\alpha\beta\delta}{K_{28}} \qquad \kappa_{PKC} = \frac{0P}{K_{10}} + \frac{\alpha+\alpha'}{K_{13}} + \frac{\alpha\beta}{K_{16}} + \frac{\alpha\beta\gamma}{K_{19}}$$

$$\kappa_{PP1} = \frac{\alpha}{K_2} + \frac{\alpha'}{K_{2,fast}} + \frac{\alpha\beta}{K_5} + \frac{\alpha\beta\gamma}{K_8} + \frac{\delta}{K_{11}} + \frac{\alpha\delta}{K_{14}} + \frac{\alpha\delta}{K_{23}} + \frac{\alpha\beta\delta}{K_{17}} + \frac{\alpha\beta\delta}{K_{26}} + \frac{\alpha\beta\gamma\delta}{K_{20}} + \frac{\alpha\beta\gamma\delta}{K_{29}}$$

$$\kappa_{PP2A} = \frac{\alpha}{K_3} + \frac{\alpha'}{K_{3,fast}} + \frac{\alpha\beta}{K_6} + \frac{\alpha\beta\gamma}{K_9} + \frac{\delta}{K_{12}} + \frac{\alpha\delta}{K_{15}} + \frac{\alpha\delta}{K_{24}} + \frac{\alpha\beta\delta}{K_{18}} + \frac{\alpha\beta\delta}{K_{27}} + \frac{\alpha\beta\gamma\delta}{K_{21}} + \frac{\alpha\beta\gamma\delta}{K_{30}}$$

Rate laws

PKA

$$v_1 = \frac{k_1 \cdot PKA \cdot 0P}{K_1 \cdot \left(1 + \kappa_{PKA} - \frac{0P}{K_1}\right) + 0P} \qquad v_4 = \frac{k_4 \cdot PKA \cdot \alpha}{K_4 \cdot \left(1 + \kappa_{PKA} - \frac{\alpha}{K_4}\right) + \alpha} \qquad v_{4,2} = \frac{k_{4,2} \cdot PKA \cdot \alpha'}{K_{4,2} \cdot \left(1 + \kappa_{PKA} - \frac{\alpha'}{K_{4,2}}\right) + \alpha'}$$

$$v_7 = \frac{k_7 \cdot PKA \cdot \delta}{K_7 \cdot \left(1 + \kappa_{PKA} - \frac{\delta}{K_7}\right) + \delta} \qquad v_{22} = \frac{k_{22} \cdot PKA \cdot \alpha\beta}{K_{22} \cdot \left(1 + \kappa_{PKA} - \frac{\alpha\beta}{K_{22}}\right) + \alpha\beta} \qquad v_{25} = \frac{k_{25} \cdot PKA \cdot \alpha\delta}{K_{25} \cdot \left(1 + \kappa_{PKA} - \frac{\alpha\delta}{K_{25}}\right) + \alpha\delta}$$

$$v_{28} = \frac{k_{28} \cdot PKA \cdot \alpha\beta\delta}{K_{28} \cdot \left(1 + \kappa_{PKA} - \frac{\alpha\beta\delta}{K_{28}}\right) + \alpha\beta\delta}$$

PKC

$$v_{10} = \frac{k_{10} \cdot PKC \cdot 0P}{K_{10} \cdot \left(1 + \kappa_{PKC} - \frac{0P}{K_{10}}\right) + 0P} \qquad v_{13} = \frac{k_{13} \cdot PKC \cdot \alpha}{K_{13} \cdot \left(1 + \kappa_{PKC} - \frac{\alpha}{K_{13}}\right) + \alpha} \qquad v_{13,2} = \frac{k_{13,2} \cdot PKC \cdot \alpha'}{K_{13,2} \cdot \left(1 + \kappa_{PKC} - \frac{\alpha'}{K_{13,2}}\right) + \alpha'}$$

$$v_{16} = \frac{k_{16} \cdot PKC \cdot \alpha\beta}{K_{16} \cdot \left(1 + \kappa_{PKC} - \frac{\alpha\beta}{K_{16}}\right) + \alpha\beta} \qquad v_{19} = \frac{k_{19} \cdot PKC \cdot \alpha\beta\gamma}{K_{19} \cdot \left(1 + \kappa_{PKC} - \frac{\alpha\beta\gamma}{K_{19}}\right) + \alpha\beta\gamma}$$

RSK2

$$v_{31} = \frac{k_{31} \cdot RSK2 \cdot 0P}{K_{31} + \frac{\delta}{K_{32}} + 0P} \qquad v_{32} = \frac{k_{32} \cdot RSK2 \cdot \delta}{K_{32} + \frac{0P}{K_{31}} + \delta}$$

PP1

$$v_2 = \frac{k_2 \cdot PP1 \cdot \alpha}{K_2 \cdot \left(1 + \kappa_{PP1} - \frac{\alpha}{K_2}\right) + \alpha} \qquad v_{2,fast} = \frac{k_{2,fast} \cdot PP1 \cdot \alpha'}{K_{2,fast} \cdot \left(1 + \kappa_{PP1} - \frac{\alpha'}{K_{2,fast}}\right) + \alpha'} \qquad v_5 = \frac{k_5 \cdot PP1 \cdot \alpha\beta}{K_5 \cdot \left(1 + \kappa_{PP1} - \frac{\alpha\beta}{K_5}\right) + \alpha\beta}$$

$$v_8 = \frac{k_8 \cdot PP1 \cdot \alpha\beta\gamma}{K_8 \cdot \left(1 + \kappa_{PP1} - \frac{\alpha\beta\gamma}{K_8}\right) + \alpha\beta\gamma} \qquad v_{11} = \frac{k_{11} \cdot PP1 \cdot \delta}{K_{11} \cdot \left(1 + \kappa_{PP1} - \frac{\delta}{K_{11}}\right) + \delta} \qquad v_{14} = \frac{k_{14} \cdot PP1 \cdot \alpha\delta}{K_{14} \cdot \left(1 + \kappa_{PP1} - \frac{\alpha\delta}{K_{14}}\right) + \alpha\delta}$$

$$v_{17} = \frac{k_{17} \cdot PP1 \cdot \alpha\beta\delta}{K_{17} \cdot \left(1 + \kappa_{PP1} - \frac{\alpha\beta\delta}{K_{17}}\right) + \alpha\beta\delta} \qquad v_{20} = \frac{k_{20} \cdot PP1 \cdot \alpha\beta\gamma\delta}{K_{20} \cdot \left(1 + \kappa_{PP1} - \frac{\alpha\beta\gamma\delta}{K_{20}}\right) + \alpha\beta\gamma\delta} \qquad v_{23} = \frac{k_{23} \cdot PP1 \cdot \alpha\delta}{K_{23} \cdot \left(1 + \kappa_{PP1} - \frac{\alpha\delta}{K_{23}}\right) + \alpha\delta}$$

$$v_{26} = \frac{k_{26} \cdot PP1 \cdot \alpha\beta\delta}{K_{26} \cdot \left(1 + \kappa_{PP1} - \frac{\alpha\beta\delta}{K_{26}}\right) + \alpha\beta\delta} \qquad v_{29} = \frac{k_{29} \cdot PP1 \cdot \alpha\beta\gamma\delta}{K_{29} \cdot \left(1 + \kappa_{PP1} - \frac{\alpha\beta\gamma\delta}{K_{29}}\right) + \alpha\beta\gamma\delta}$$

PP2A

$v_3 = \frac{k_3 \cdot PP2A \cdot \alpha}{K_3 \cdot \left(1 + \kappa_{PP2A} - \frac{\alpha}{K_3}\right) + \alpha}$ $v_{3,fast} = \frac{k_{3,fast} \cdot PP2A \cdot \alpha\prime}{K_{3,fast} \cdot \left(1 + \kappa_{PP2A} - \frac{\alpha'}{K_{3,fast}}\right) + \alpha\prime}$ $v_6 = \frac{k_6 \cdot PP2A \cdot \alpha\beta}{K_6 \cdot \left(1 + \kappa_{PP2A} - \frac{\alpha\beta}{K_6}\right) + \alpha\beta}$

$v_9 = \frac{k_9 \cdot PP2A \cdot \alpha\beta\gamma}{K_9 \cdot \left(1 + \kappa_{PP2A} - \frac{\alpha\beta\gamma}{K_0}\right) + \alpha\beta\gamma}$ $v_{12} = \frac{k_{12} \cdot PP2A \cdot \delta}{K_{12} \cdot \left(1 + \kappa_{PP2A} - \frac{\delta}{K_{12}}\right) + \delta}$ $v_{15} = \frac{k_{15} \cdot PP2A \cdot \alpha\delta}{K_{15} \cdot \left(1 + \kappa_{PP2A} - \frac{\alpha\delta}{K_{15}}\right) + \alpha\delta}$

$v_{18} = \frac{k_{18} \cdot PP2A \cdot \alpha\beta\delta}{K_{18} \cdot \left(1 + \kappa_{PP2A} - \frac{\alpha\beta\delta}{K_{18}}\right) + \alpha\beta\delta}$ $v_{21} = \frac{k_{21} \cdot PP2A \cdot \alpha\beta\gamma\delta}{K_{21} \cdot \left(1 + \kappa_{PP2A} - \frac{\alpha\beta\gamma\delta}{K_{21}}\right) + \alpha\beta\gamma\delta}$ $v_{24} = \frac{k_{24} \cdot PP2A \cdot \alpha\delta}{K_{24} \cdot \left(1 + \kappa_{PP2A} - \frac{\alpha\delta}{K_{24}}\right) + \alpha\delta}$

$v_{27} = \frac{k_{27} \cdot PP2A \cdot \alpha\beta\delta}{K_{27} \cdot \left(1 + \kappa_{PP2A} - \frac{\alpha\beta\delta}{K_{27}}\right) + \alpha\beta\delta}$ $v_{30} = \frac{k_{30} \cdot PP2A \cdot \alpha\beta\gamma\delta}{K_{30} \cdot \left(1 + \kappa_{PP2A} - \frac{\alpha\beta\gamma\delta}{K_{30}}\right) + \alpha\beta\gamma\delta}$

Isomerization

$v_{iso,F} = k_{iso,F} \cdot \alpha'$ $v_{iso,R} = k_{iso,R} \cdot \alpha$

ODEs

$$\frac{d}{dt} 0P = v_2 + v_{2,fast} + v_3 + v_{3,fast} + v_{11} + v_{12} - v_1 - v_{10} - v_{31}$$

$$\frac{d}{dt} \alpha = v_1 + v_{31} - v_2 - v_3 - v_4 - v_{13} + v_{iso,F} - v_{iso,R}$$

$$\frac{d}{dt} \alpha' = v_5 + v_6 + v_{14} + v_{15} - v_{iso,F} + v_{iso,R} - v_{2,fast} - v_{3,fast} - v_{4,2} - v_{13,2}$$

$$\frac{d}{dt} \alpha\beta = v_4 + v_{4,2} + v_8 + v_9 + v_{17} + v_{18} - v_5 - v_6 - v_7 - v_{16}$$

$$\frac{d}{dt} \alpha\beta\gamma = v_7 + v_{20} + v_{21} - v_8 - v_9 - v_{19}$$

$$\frac{d}{dt} \delta = v_{10} + v_{23} + v_{24} - v_{11} - v_{12} - v_{22} - v_{32}$$

$$\frac{d}{dt} \alpha\delta = v_{13} + v_{13,2} + v_{22} + v_{26} + v_{27} + v_{32} - v_{14} - v_{15} - v_{23} - v_{24} - v_{25}$$

$$\frac{d}{dt} \alpha\beta\delta = v_{16} + v_{25} + v_{29} + v_{30} - v_{17} - v_{18} - v_{26} - v_{27} - v_{28}$$

$$\frac{d}{dt} \alpha\beta\gamma\delta = v_{19} + v_{28} - v_{20} - v_{21} - v_{29} - v_{30}$$

Model 5 (tQSSA)

Like Model 4 except that reaction rates of enzyme catalyzed reations follow the tQSSA rate law $v_j = \frac{k_j \cdot e_T \cdot s_j}{(K_j + e_T)(1 + \sum_{i=1}^{n} \frac{s_i}{K_i + e_T})}$ and competition terms κ_{ENZYME} are not needed anymore. ODEs remain the same.
