# Supplementary material for: Cardiac myosin binding protein-C phosphorylation as a function of multiple protein kinase and phosphatase activities
Source: Nat Commun. 2024 Jun 14;15:5111. doi: 10.1038/s41467-024-49408-5 (PMC11178824; doi:10.1038/s41467-024-49408-5)
Supplement: Supplementary file 2 — Reporting Summary [file 41467_2024_49408_MOESM2_ESM.pdf]

nature portfolio

Corresponding author(s): Daniel Koch
Last updated by author(s): May 24, 2024

Reporting Summary

Nature Portfolio wishes to improve the reproducibility of the work that we publish. This form provides structure for consistency and transparency in reporting. For further information on Nature Portfolio policies, see our Editorial Policies and the Editorial Policy Checklist.

Statistics

For all statistical analyses, confirm that the following items are present in the figure legend, table legend, main text, or Methods section.

n/a	Confirmed	
☐	☒	The exact sample size (*n*) for each experimental group/condition, given as a discrete number and unit of measurement
☐	☒	A statement on whether measurements were taken from distinct samples or whether the same sample was measured repeatedly
☐	☒	The statistical test(s) used AND whether they are one- or two-sided<br>*Only common tests should be described solely by name; describe more complex techniques in the Methods section.*
☒	☐	A description of all covariates tested
☐	☒	A description of any assumptions or corrections, such as tests of normality and adjustment for multiple comparisons
☐	☒	A full description of the statistical parameters including central tendency (e.g. means) or other basic estimates (e.g. regression coefficient) AND variation (e.g. standard deviation) or associated estimates of uncertainty (e.g. confidence intervals)
☐	☒	For null hypothesis testing, the test statistic (e.g. *F*, *t*, *r*) with confidence intervals, effect sizes, degrees of freedom and *P* value noted<br>*Give P values as exact values whenever suitable.*
☒	☐	For Bayesian analysis, information on the choice of priors and Markov chain Monte Carlo settings
☒	☐	For hierarchical and complex designs, identification of the appropriate level for tests and full reporting of outcomes
☒	☐	Estimates of effect sizes (e.g. Cohen's *d*, Pearson's *r*), indicating how they were calculated

Our web collection on statistics for biologists contains articles on many of the points above.

Software and code

Policy information about availability of computer code

Data collection	Custom code written in python. Details and exact versions of the python packages used are given in the README and the github repository (https://github.com/KochLabCode/cMyBP-C-phosphorylation/).
Data analysis	Nanotemper MO.affinity analysis v2.2.4, Graphpad Prism 9 and custom code written in python. Details and exact versions of the python packages used are given in the README and the github repository (https://github.com/KochLabCode/cMyBP-C-phosphorylation/).

For manuscripts utilizing custom algorithms or software that are central to the research but not yet described in published literature, software must be made available to editors and reviewers. We strongly encourage code deposition in a community repository (e.g. GitHub). See the Nature Portfolio guidelines for submitting code & software for further information.

Data

Policy information about availability of data

All manuscripts must include a data availability statement. This statement should provide the following information, where applicable:

- Accession codes, unique identifiers, or web links for publicly available datasets
- A description of any restrictions on data availability
- For clinical datasets or third party data, please ensure that the statement adheres to our policy

All experimental data are available in the source data file except the mass spectometry data, which is available at the massIVE repository under accession code MSV000091995 [https://massive.ucsd.edu/ProteoSAFe/dataset.jsp?accession=MSV000091995].

Research involving human participants, their data, or biological material

Policy information about studies with human participants or human data. See also policy information about sex, gender (identity/presentation), and sexual orientation and race, ethnicity and racism.

Reporting on sex and gender	Data from human donors and heart failure patients was taken as the published mean +/- SEM of cMyBP-C phosphorylation reported in Copeland et al. 2010 without consideration of further characteristics. For details regarding sex, gender, race, ethnicity and other groupings, characteristics, recruitment and ethics see Copeland, O. et al. Analysis of cardiac myosin binding protein-C phosphorylation in human heart muscle. Journal of Molecular and Cellular Cardiology 49, 1003–1011 (2010). DOI: 10.1016/j.yjmcc.2010.09.007.
Reporting on race, ethnicity, or other socially relevant groupings	Data from human donors and heart failure patients was taken as the published mean +/- SEM of cMyBP-C phosphorylation reported in Copeland et al. 2010 without consideration of further characteristics. For details regarding sex, gender, race, ethnicity and other groupings, characteristics, recruitment and ethics see Copeland, O. et al. Analysis of cardiac myosin binding protein-C phosphorylation in human heart muscle. Journal of Molecular and Cellular Cardiology 49, 1003–1011 (2010). DOI: 10.1016/j.yjmcc.2010.09.007.
Population characteristics	Data from human donors and heart failure patients was taken as the published mean +/- SEM of cMyBP-C phosphorylation reported in Copeland et al. 2010 without consideration of further characteristics. For details regarding sex, gender, race, ethnicity and other groupings, characteristics, recruitment and ethics see Copeland, O. et al. Analysis of cardiac myosin binding protein-C phosphorylation in human heart muscle. Journal of Molecular and Cellular Cardiology 49, 1003–1011 (2010). DOI: 10.1016/j.yjmcc.2010.09.007.
Recruitment	Data from human donors and heart failure patients was taken as the published mean +/- SEM of cMyBP-C phosphorylation reported in Copeland et al. 2010 without consideration of further characteristics. For details regarding sex, gender, race, ethnicity and other groupings, characteristics, recruitment and ethics see Copeland, O. et al. Analysis of cardiac myosin binding protein-C phosphorylation in human heart muscle. Journal of Molecular and Cellular Cardiology 49, 1003–1011 (2010). DOI: 10.1016/j.yjmcc.2010.09.007.
Ethics oversight	Data from human donors and heart failure patients was taken as the published mean +/- SEM of cMyBP-C phosphorylation reported in Copeland et al. 2010 without consideration of further characteristics. For details regarding sex, gender, race, ethnicity and other groupings, characteristics, recruitment and ethics see Copeland, O. et al. Analysis of cardiac myosin binding protein-C phosphorylation in human heart muscle. Journal of Molecular and Cellular Cardiology 49, 1003–1011 (2010). DOI: 10.1016/j.yjmcc.2010.09.007.

Note that full information on the approval of the study protocol must also be provided in the manuscript.

Field-specific reporting

Please select the one below that is the best fit for your research. If you are not sure, read the appropriate sections before making your selection.

☒ Life sciences ☐ Behavioural & social sciences ☐ Ecological, evolutionary & environmental sciences

For a reference copy of the document with all sections, see nature.com/documents/nr-reporting-summary-flat.pdf

Life sciences study design

All studies must disclose on these points even when the disclosure is negative.

Sample size	No sample size calculations were performed. The sample size used for each experiment was chose to be statistically adequate to describe and interpret the results while minimizing materials/resources used in the study (generally we chose between n=1 (e.g. antibody validation) to n=100 (e.g. for parameter estimation simulations).
Data exclusions	No data were excluded from analysis.
Replication	All experiments included in the paper were repeated multiple times. All replicates are independent experiments (i.e. each datapoint represents a different sample). All attempts at replicating the data were successful.
Randomization	Not relevant to current study
Blinding	Not relevant to current study

Reporting for specific materials, systems and methods

We require information from authors about some types of materials, experimental systems and methods used in many studies. Here, indicate whether each material, system or method listed is relevant to your study. If you are not sure if a list item applies to your research, read the appropriate section before selecting a response.

Materials & experimental systems

n/a	Involved in the study
[ ]	[x] Antibodies
[x]	[ ] Eukaryotic cell lines
[x]	[ ] Palaeontology and archaeology
[x]	[ ] Animals and other organisms
[x]	[ ] Clinical data
[x]	[ ] Dual use research of concern
[x]	[ ] Plants

Methods

n/a	Involved in the study
[x]	[ ] ChIP-seq
[x]	[ ] Flow cytometry
[x]	[ ] MRI-based neuroimaging

Antibodies

Antibodies used	cMyBP-C pS279, pS288 and pS279 antibodies were produced and affinity purified by ProSci Inc (CA) as described by Sadayyapan et al. 2009 (doi: 10.1161/CIRCULATIONAHA.108.798983) using the corresponding human (phospho)peptide sequences AFRRT(pS)LAGAG, GAGRRT(pS)DSHEDA and LKKRD(pS)FRRDS.
Validation	Specificity of generated antibodies was confimed by ProSci Inc. via ELISA using the corresponding phospho-peptides (data are in the source data file) and by us via Western-blot (Supplementary Figure 31).
